# A novel strategy for delivering Niemann‐Pick type C2 proteins across the blood–brain barrier using the brain endothelial‐specific AAV‐BR1 virus

**DOI:** 10.1111/jnc.15621

**Published:** 2022-05-25

**Authors:** Charlotte Laurfelt Munch Rasmussen, Eva Hede, Lisa Juul Routhe, Jakob Körbelin, Steinunn Sara Helgudottir, Louiza Bohn Thomsen, Markus Schwaninger, Annette Burkhart, Torben Moos

**Affiliations:** ^1^ Neurobiology Research and Drug Delivery, Department of Health Science and Technology Aalborg University Aalborg Denmark; ^2^ Department of Oncology, Hematology and Bone Marrow Transplantation University Medical Center Hamburg Germany; ^3^ Institute for Experimental and Clinical Pharmacology and Toxicology University of Lübeck Lübeck Germany

**Keywords:** AAV2, AAV‐BR1, blood–brain barrier, Niemann Pick disease type C2, viral gene therapy

## Abstract

Treating central nervous system (CNS) diseases is complicated by the incapability of numerous therapeutics to cross the blood–brain barrier (BBB), mainly composed of brain endothelial cells (BECs). Genetically modifying BECs into protein factories that supply the CNS with recombinant proteins is a promising approach to overcome this hindrance, especially in genetic diseases, like Niemann Pick disease type C2 (NPC2), where both CNS and peripheral cells are affected. Here, we investigated the potential of the BEC‐specific adeno‐associated viral vector (AAV‐BR1) encoding NPC2 for expression and secretion from primary BECs cultured in an *in vitro* BBB model with mixed glial cells, and in healthy BALB/c mice. Transduced primary BECs had significantly increased NPC2 gene expression and secreted NPC2 after viral transduction, which significantly reversed cholesterol deposition in NPC2 deficient fibroblasts. Mice receiving an intravenous injection with AAV‐BR1‐NCP2‐eGFP were sacrificed 8 weeks later and examined for its biodistribution and transgene expression of eGFP and NPC2. AAV‐BR1‐NPC2‐eGFP was distributed mainly to the brain and lightly to the heart and lung, but did not label other organs including the liver. eGFP expression was primarily found in BECs throughout the brain but occasionally also in neurons suggesting transport of the vector across the BBB, a phenomenon also confirmed *in vitro*. NPC2 gene expression was up‐regulated in the brain, and recombinant NPC2 protein expression was observed in both transduced brain capillaries and neurons. Our findings show that AAV‐BR1 transduction of BECs is possible and that it may denote a promising strategy for future treatment of NPC2.

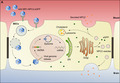

## Graphical abstract

Brain endothelial cells (BECs), denoting the blood–brain barrier (BBB), can be specifically transduced both *in vitro* and in vivo following intravenous administration of the AAV‐BR1 vector, which onsets the synthesis of recombinant NPC2 protein from the BECs. *In vitro* transduction of BECs resulted in a bidirectional secretion of NPC2 in sufficient amount to reverse pathological cholesterol accumulation in NPC2 deficient fibroblasts. In healthy mice, high viral concentrations were primarily observed in the brain capillaries, with resulting production of recombinant NPC2. AAV‐BR1 based gene therapy specifically towards the BBB denotes a promising strategy to treat Niemann Pick disease type C2.

**Abbreviations**
AAV‐BR1: brain endothelial cell‐specific adeno‐associated virus 2
BBB: blood–brain barrier
BCSFB: blood‐cerebrospinal fluid barrier
BECs: brain endothelial cells
BIIC: baculovirus‐infected insect cell
CD11b: cluster of differentiation molecule 11b
CLD5: claudin 5
CNS: central nervous system
CTRL: control
DMSO: dimethyl sulfoxide
eGFP: enhanced green fluorescent protein
FCS: fetal calf serum
GFAP: glial fibrillary acidic protein
*GLUT1*
: glucose transporter 1
LSD: lysosomal storage disease
mBECs: mouse brain endothelial cells
NeuN: neuronal nuclei antigen
NPC: Niemann Pick disease type C
PBS: phosphate‐buffered saline
PPBS: potassium containing phosphate‐buffered saline
TEER: trans‐endothelial electrical resistance
vg: viral genomes
ZO1: Zonula occludens

## 1 INTRODUCTION

**FIGURE 1:**
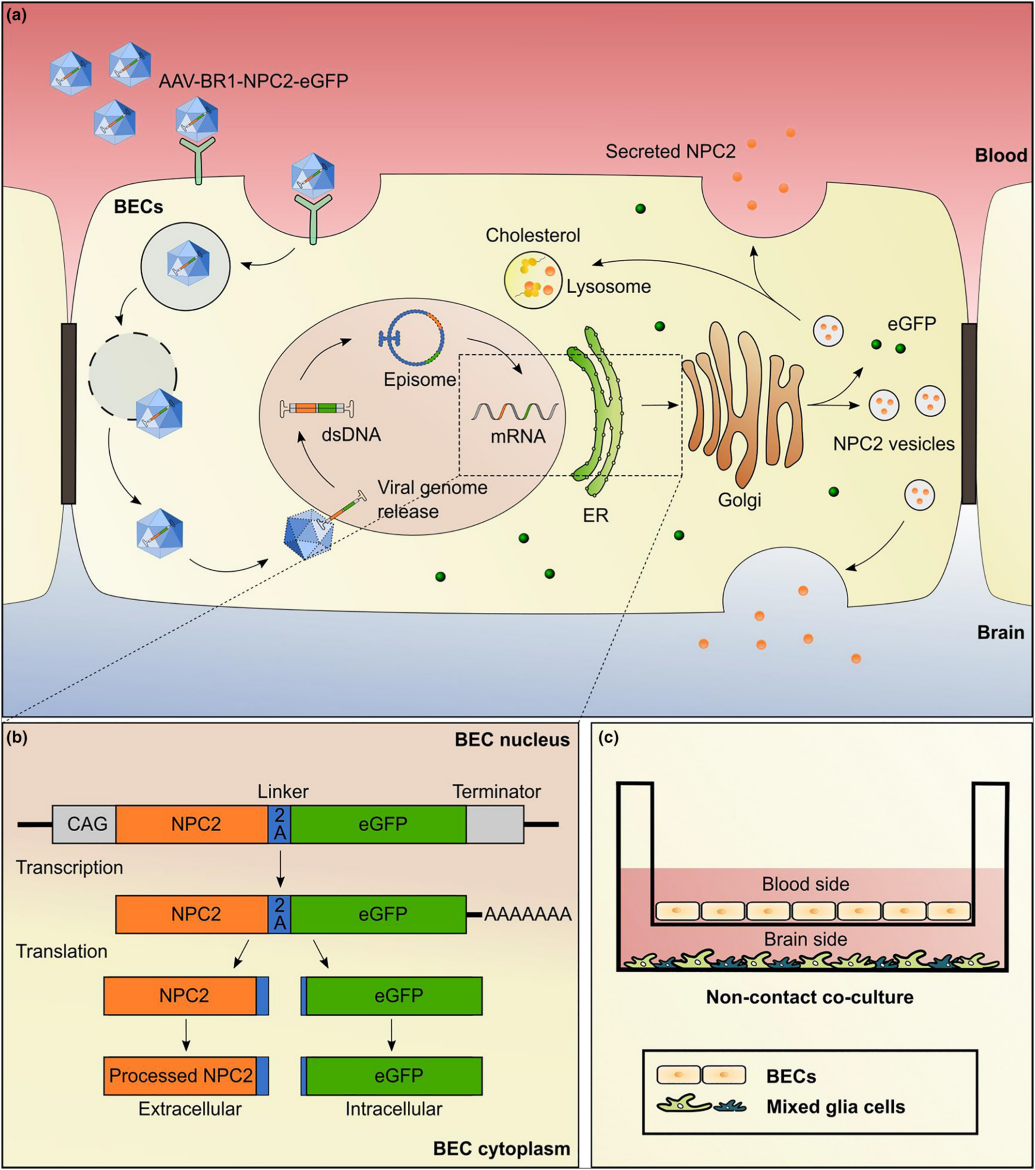
Schematic presentation of the strategy to genetically modify brain endothelial cells (BECs) to enable secretion of Niemann Pick type C2 protein (NPC2). (a) The brain endothelial cell‐specific adeno‐associated virus (AAV‐BR1) vector encoding the NPC2 and enhanced green fluorescent protein (eGFP) genes enter the BECs through receptor‐mediated endocytosis via an unknown receptor, leading to the release of the partially degraded capsid as a result of decreasing pH. After the endosomal escape, the viral particle is transported to the nucleus where the viral double‐stranded DNA (ds‐DNA) is released to induce episomal transgene expression by transcription to mRNA. Transcribed mRNA is transported to the endoplasmatic reticulum (ER) for translation, during which a ribosomal skip is induced by the 2A linker sequence, resulting in the expression of NPC2 and eGFP as two separate proteins (b). NPC2 is subsequently modified in the Golgi apparatus from which it will be transported via the mannose‐6‐phosphate receptor either to the lysosomes to facilitate cholesterol transport, or to vesicles for secretion toward either the bloodstream or the brain parenchyma. eGFP will accumulate within the cells and is, therefore, a marker for cell transduction and transgene expression (a + b). The bi‐directional secretion of NPC2 will thus theoretically supply NPC2 to NPC2 deficient cells both inside and outside of the central nervous system. (c) An *in vitro* blood–brain barrier model using primary mouse BECs in non‐contact co‐culture with primary mouse glial cells.

The restrictions of the blood–brain barrier (BBB), limiting the passage of harmful molecules from the circulation, remains a major obstacle for drug delivery to the brain (Pardridge, 2020) and consequently, demands improved drug delivery strategies (Pardridge, 2012; Terstappen et al., 2021). The BBB is composed of brain endothelial cells (BECs) lining the capillary wall supported by pericytes and astrocytic end‐feet (Abbott et al., 2009; Alvarez et al., 2011). It controls the entry of molecules into the brain to maintain a stable microenvironment, crucial for proper neuronal function (Abbott et al., 2009; Alvarez et al., 2011; Armulik et al., 2010; Daneman et al., 2010; Misje et al. 2010). Several different methods have been explored to overcome the hindrance of the BBB, for example, temporarily increasing BBB permeability using hyperosmolar solutes and focused ultrasound, directly infusing compounds into the brain, and exploiting targeting to existing mechanisms for nutrient transport at the BBB. Yet, these efforts are often associated with a risk of serious adverse events, limited distribution within the brain, or low brain specificity, respectively (Assmann et al., 2016; Johnsen & Moos, 2016; Johnsen et al., 2017; McMahon et al., 2019; Tajes et al., 2014). Other strategies used to enhance the BBB crossing includes engineering adeno‐associated virus (AAV) vectors, for example, AAV‐PHP.eB and AAV‐PHP.B, but these vectors are not brain permissive in all mouse strains, which limits the translation beyond mouse models (Matsuzaki et al., 2019; Mathiesen et al., 2020). An alternative approach to bypass the restrictive functions of the BBB is gene therapy to the BECs to induce protein synthesis and secretion (Figure 1a) (Burkhart et al., 2017; Chen et al., 2009; Hede et al., 2020; Jiang et al., 2003; Thomsen et al., 2011). While succeeding in delivering proteins to the brain, this approach potentially also carries the risk of adverse events since proteins secreted from BECs into the peripheral circulation may result in off‐target effects (Burkhart et al., 2017; Hede et al., 2020). Nevertheless, the bi‐directional secretion from the BECs denotes a relevant strategy in diseases with a global impact on body function because of a systemic lack of a functioning protein, as occurring in the group of inherited metabolic diseases, known as lysosomal storage diseases (LSD), characterized by lysosomal dysfunctions because of protein and enzyme deficiencies (Ashtari et al., 2016).

Niemann Pick disease type C (NPC), is an LSD caused by deficiency of one of two lysosomal cholesterol transporter proteins, referred to as NPC1 or NPC2 (Storch & Xu, 2009; Vanier & Millat, 2003). It is a rare autosomal recessive disease with an estimated incidence of 1:100,000 with 5% of the cases caused by a deficiency in NPC2 and 95% in NPC1. NPC is fatal in almost all cases presenting a variety of both systemic and neurological symptoms starting at infancy (Vanier, 2010). The severity of the disease is associated with the type of the genetic mutation, but approximately 10% of the patients die before the age of 6 months, while most patients live up until the age of 30 years with progressive symptom development (Vanier & Millat, 2003; Wraith et al., 2009). At present, the only approved treatment option for NPC is the non‐curative glucosylceramide synthase inhibitor, Miglustat, which increases survival by delaying the onset of symptoms (Patterson & Walkley, 2017; Vanier, 2010).

The functional lack of either of the two cholesterol transporter proteins causing NPC includes NPC1, a large membrane‐bound protein located within the lysosomal membranes, and the much smaller and soluble NPC2 protein that is mainly located within the lysosomal core. Secreted NPC2 is internalized by adjacent cells via the cation‐independent mannose‐6‐phosphate receptor (Sands & Davidson, 2006; Storch & Xu, 2009). The ability to internalize extracellular lysosomal proteins has been exploited as enzyme replacement therapies in other LSDs caused by a deficiency in soluble lysosomal proteins (Beck, 2009; Dogbevia et al., 2020; Fratantoni et al., 1968; Lim‐Melia & Kronn, 2009; Rohrbach & Clarke, 2007). Systemic administration of NPC2 protein improved systemic symptoms but was unable to improve neurological symptoms probably because of the restraints of the BBB obstructing NPC2 transport into the brain (Nielsen et al., 2011).

The strategy of using gene therapy to the BBB to induce secretion of therapeutic proteins previously underwent thorough examinations *in vitro* taking non‐viral approaches (Burkhart et al., 2015, 2017; Hede et al., 2020; Thomsen et al., 2011). In the current study, we undertook a viral approach using the BEC‐specific adeno‐associated viral vector (AAV‐BR1) previously shown to specifically target BECs after intravenous injection (Dogbevia et al., 2020; Körbelin et al., 2016). The BEC‐targeted AAV capsid mutant “AAV‐BR1” has previously been selected after several rounds of in vivo screening of a random AAV2 display peptide library in mice (Körbelin et al., 2016). In this study, the AAV‐BR1 vector was used to enable the production of two separate proteins, that is, enhanced green fluorescent protein (eGFP) and NPC2 of which eGFP would accumulate within the transduced cells, indicating cellular transduction, while NPC2 was destined for both accumulation in BECs lysosomes and secretion further into the brain to allow uptake by neighboring non‐transduced cells (Figure 1a,b).

## 2 MATERIALS AND METHODS

### 2.1 Vector production

Recombinant AAV‐BR1‐NPC2‐eGFP vectors were produced using the baculovirus expression vector system, which requires the infection of insect cells with two different baculoviruses; one carrying the AAV *rep* and *cap* genes and one carrying the gene of interest flanked by AAV‐inverted terminal repeat regions (Chen, 2008; Kohlbrenner et al., 2005; Urabe et al., 2002; Wasilko et al., 2009). The generation of recombinant baculoviruses was based on the Bac‐to‐Bac™ Baculovirus Expression System (Thermo Fisher Scientific, #10359‐016). The plasmids used for the generation of recombinant baculovirus stocks were prepared by cloning the desired genes into a pFASTBAC™ donor plasmid. These included a pFB‐AAV‐BR1‐Rep/Cap (Körbelin et al., 2016), containing an AAV2 *rep* gene and a BEC‐specific AAV‐BR1 *cap* gene cloned into a pFASTBAC™ Dual vector (Thermo Fisher Scientific, #10712‐024), and a pFB‐CAG‐mNPC2‐2A‐eGFP containing a CAG promoter, a mouse NPC2 gene linked to an eGFP reporter and AAV2 inverted terminal repeat regions cloned into a pFASTBAC™ 1 vector (Thermo Fisher Scientific, #10359‐016). The NPC2 and eGFP genes were linked via a 2A linker, inducing a ribosomal skip during translation, which results in the production of NPC2 and eGFP as two separate proteins for every transcript (Figure 1b; Donnelly et al., 2001; Liu et al., 2017).

DH10BAC™ *Escherichia coli* (Thermo Fisher Scientific, #10361012), containing the baculovirus genome for bacterial transposition, were transformed with pFB‐AAV‐BR1‐Rep/Cap or pFB‐CAG‐mNPC2‐2A‐eGFP by heat shock, inducing a site‐specific transposition of the pFASTBAC™ expression cassette into the bacmid Tn7 target site within the DH10BAC™ *E. coli* to create an expression bacmid (Luckow et al., 1993). Transformed DH10BACTM *E. coli* were then reconstituted in SOC medium for 4 h at 37°C before being plated on selection plates and incubated for 72 h to induce the formation of blue/white colonies. Positive white colonies were subsequently amplified under selection before purifying the expression bacmids.

Baculovirus‐infected insect cell (BIIC) stocks were created as previously described for large‐scale production of recombinant AAVs (Wasilko et al., 2009). Initially, Sf9 insect cells were seeded at a density of 1.5 × 10^5^ cells/cm^2^ in Insect‐XPRESS™ Insect Culture Media (Lonza, #12‐730Q) supplemented with 10 mg/L gentamicin and left to adhere for 30 min at 27°C. The Sf9 cells were then transfected with the purified expression bacmid DNA containing the AAV‐BR1‐Rep/Cap genes or the mNPC2‐2A‐eGFP genes using TransIT Transfection Reagent (Mirus Bio, Kem‐En‐Tec Nordic, #MIR 6104) and incubated at 27°C for 72 h. Following the incubation, the cell supernatant containing free baculovirus was collected and used to infect new Sf9 cells, which were then incubated for 48 h at 27°C to amplify the baculoviruses. BIIC stocks were then harvested, centrifuged, and cryopreserved at −80°C in insect cryomedium [1:1 fresh and conditioned Insect‐XPRESS™ Insect Culture Media supplemented with 7.5% dimethyl sulfoxide (DMSO)] in aliquots with concentrations of approximately 3 × 10^7^ cells/ml. Before the large‐scale production of the recombinant AAV2s, the BIIC stocks containing AAV‐BR1‐Rep/Cap or the mNPC2‐2A‐eGFP genes were tested to determine the optimal concentration of the BIIC stocks for the production of recombinant AAV2s by co‐infection of Sf9 cells. Initially, the AAV‐BR1‐Rep/Cap BIIC stock was tested by infecting Sf9 cells with various concentrations of the AAV‐BR1‐Rep/Cap BIIC stock and evaluated for the expression of capsid proteins 96 h post‐infection by western blot using mouse anti‐AAV‐VP1/VP2/VP3 antibodies (Wobus et al., 2000). Next, the mNPC2‐2A‐eGFP BIIC stock was tested by co‐infection of Sf9 cells with the determined ideal concentration of AAV‐BR1‐Rep/Cap BIIC stock and various concentrations of the mNPC2‐2A‐eGFP BIIC stock. The production of recombinant AAV‐BR1‐NPC2‐eGFP vectors was then evaluated 96 h post‐infection by quantification of the vector copy numbers using absolute qPCR. Before the qPCR analysis, viral particles were released from the insect cells by repeated freeze/thaw cycles, and unpackaged DNA was removed by digestion with 50 U/ml Benzonase Nuclease (Merck KGaA, #8263). For the determination of vector copy numbers, the FastStart Essential DNA Green Master (Merck KGaA, #06402712001 Roche) based on SYBR green was used with the Light Cycler 96 System (Roche). Primers specific for the CAG promoter were used (5′‐AAC GCC AAT AGG GAC TTT C‐3′ and 5′‐GTA GGA AAG TCC CAT AAG GTC A‐3′) in final concentrations of 0.25 μM. Viral particles were measured in 1:1000 and 1:10,000 dilutions and a corresponding plasmid DNA was used to generate a standard curve. Reactions were run with an initial denaturation at 95°C for 10 min followed by 40 cycles of denaturation at 95°C for 30 s, annealing at 67°C for 30 s, and extension at 72°C for 30 s, followed by a melt curve analysis (60–97°C, 0.1°C/s).

Recombinant AAV‐BR1‐NPC2‐eGFP vectors were finally produced on a large scale by co‐infection of Sf9 cells with the determined optimal concentrations of AAV‐BR1‐Rep/Cap and mNPC2‐2A‐eGFP BIIC stocks. For large‐scale production, Sf9 cells were seeded in suspension at a density of 1.5 × 10^6^ cells/ml and incubated at 27°C in Insect‐XPRESS™ Insect Culture Media (Lonza, #12‐730Q) supplemented with 10 mg/L gentamicin at 110 rounds per minute. Following co‐infection with BIIC stocks, the Sf9 cells were incubated for 96 h, and the cells were pelleted by centrifugation. Viral particles were harvested from the supernatant by overnight PEG‐8000/NaCl precipitation at 4°C followed by resuspension in phosphate‐buffered saline (PBS) with 1 mM MgCl2, and 2.5 mM KCl (PBS‐MK), while viral particles within the cells were resuspended in PBS‐MK and released by repeated freeze/thaw cycles. Finally, the suspension containing all harvested viral particles was digested with 50 U/ml Benzonase Nuclease, to remove potentially unpackaged DNA. Harvested AAV‐BR1‐NPC2‐eGFP vectors were purified by iodixanol gradient ultracentrifugation as described previously (Zolotukhin et al., 1999) to ensure a pure formulation prior to in vivo administration. Briefly, the harvested AAV‐BR1‐NPC2‐eGFP vectors were added to a QuickSeal ultracentrifugation tube (Beckman Coulter, #344322) after which 15%, 25%, 40%, and 54% OptiPrep™ iodixanol (Progen, Nordic BioSite, **#**1114542) dissolved in PBS‐MK was gently placed below. The 25% and 54% iodixanol solutions were stained with phenol red to be able to distinguish the layers from each other. The gradient was then ultracentrifuged at 230,000 × *g* for 70 min at 18°C. Purified AAV‐BR1‐NPC2‐eGFP vectors were finally extracted from the 40% iodixanol layer, and the viral titer was determined at the genomic level by quantification of the vector copy numbers using absolute qPCR as described above for testing the BIIC stocks.

Recombinant AAV‐BR1‐Luciferase vectors were produced as described above, based on the same pFB‐AAV‐BR1‐Rep/Cap (Körbelin et al., 2016), containing the AAV2 *rep* gene and the BEC‐specific AAV‐BR1 *cap* gene and pFB‐CAG‐Luciferase containing the Luciferase gene, the CAG promoter, and AAV2 inverted terminal repeat regions cloned into the pFASTBAC™ 1 vector (Thermo Fisher Scientific, #10359‐016).

### 2.2 Primary *in vitro* BBB model

Primary cultures of mouse brain endothelial cells (mBECs) were obtained from 8‐week‐old BALB/c mice as described previously (Thomsen et al., 2017, 2021). A total of 30 female mice, corresponding to two isolations of mBECs (15 brains were pooled for each isolation before plating), were used in this study. All animals were supplied by Janvier Lab, for housing conditions see the section regarding the in vivo studies. On the day of mBECs isolation, all animals were anesthetized using isoflurane before they were sacrificed by decapitation. After isolation, primary mBECs were either cultured directly or frozen as microvessels in fetal calf serum (FCS) (Life Technology, #10270) supplemented with 10% DMSO (Merck KGaA, #D2650) for later use. Before culturing of primary mBECs, surface areas were coated twice with collagen IV (Merck KGaA, #C5533) and fibronectin (Merck KGaA, #F1141), and the mBECs were maintained in mBECs media consisting of DMEM‐F12 (Life Technology, #31331) supplemented with 10% plasma‐derived bovine serum (First Link, #60‐00‐810), 10 μg/ml Insulin transferrin sodium selenite (Merck KGaA, #11074547001), 10 μg/ml gentamicin sulfate (Lonza, #17‐518Z), and 1 ng/μl basic fibroblast growth factor (PeproTech Nordic, #100‐18B). To remove potential contamination by pericytes, 4 μg/ml puromycin (Merck KGaA, #P8833) was added to the mBECs media for the first 4 days of culturing (Calabria et al., 2006; Perrière et al., 2005), prior to co‐culturing with glial cells.

Primary mixed glial cells (consisting mainly of astrocytes and some microglia [Thomsen et al., 2017]) were obtained from 2 days old C57BL/6 mice of both sexes as described previously (Thomsen et al., 2017). A total of two mice were used in these experiments, corresponding to two isolations of primary glial cells. All surface areas for glial cells were coated with poly‐l‐lysine (Merck KGaA, #P6282) and glial cells were maintained in glia media consisting of DMEM (low Glucose) (Life Technology, #21885) supplemented with 10% FCS and 10 μg/ml gentamicin sulfate. Following isolation, primary glial cells were cultured for a minimum of 2 weeks in T75 flasks before being frozen in glial media supplemented with 20% FCS (30% in total) and 7.5% DMSO. Before co‐culturing with mBECs glial cells were thawed and seeded in 12 well plates for an additional 2 weeks without passaging to ensure a completely confluent monolayer of glial cells (P#1).

A murine *in vitro* BBB non‐contact co‐culture model was established as described previously by co‐culturing primary mBECs with primary glial cells (Figure 1c) (Thomsen et al., 2017, 2021). mBECs were seeded onto hanging culture inserts (P#1), with a transparent polyethylene terephthalate membrane and a pore diameter of 1 μm (*in vitro*, #665610), at a cell density of approximately 100 000 cells/cm^2^ to obtain a confluent monolayer the following day. Expression of tight junctions and barrier integrity were induced by the addition of 250 μM 8‐(4‐Chlorphenylthio)adenosine 3′,5′‐cyclic monophosphate sodium salt (CTP‐cAMP) (Merck KGaA, #C3912), 17.5 μM 4‐(3‐Butoxy‐4‐methoxybenzyl)imidazoline‐2‐one (RO) (Merck KGaA, #B8279) and 550 nM hydrocortisone (Merck KGaA, #H4001) to the media in the upper chamber and 550 nM hydrocortisone to the media of the lower chamber, after moving the hanging culture insert containing mBECs to the 12‐well plate containing glial cells. In the upper chamber, the media composition was based on mBECs media, while the lower contained a 1:1 mixture of mBECs media and glial conditioned media. Barrier integrity was evaluated daily by measuring trans‐endothelial electrical resistance (TEER) using a MiliCell ERS‐2 epithelial volt‐ohm Meter and an STX01 chopstick electrode (Millipore). TEER values were subsequently calculated by subtracting TEER measurements of cell‐free double‐coated hanging filter inserts from TEER measurements of co‐cultures. Finally, the difference was multiplied by the filter area (1.12 cm^2^) to express the resistance as Ω × cm^2^.

### 2.3 *In vitro* transduction assay

*In vitro* transduction was performed using the *in vitro* BBB non‐contact co‐culture model with primary mBECs and glial cells. The AAV‐BR1‐NPC2‐eGFP vector was added to the upper chamber, representing the blood side of the BBB model, to mimic the intravenous administration route in vivo. A dose of 10^10^ viral vectors was added per hanging culture insert, based on a previous study using the AAV‐BR1 vector (Körbelin et al., 2016), corresponding to a multiplicity of infection of approximately 10^5^. 24 h after virus administration, the media was changed to remove excess viral particles, and the conditioned media was stored at −80°C for subsequent quantification of residual viral vectors. TEER was measured before transduction and medium change and subsequently every 24 h to continuously evaluate the barrier integrity. The conditioned medium containing possibly secreted recombinant NPC2 was harvested 4 days post‐transduction and stored at −80°C for further analysis, while mBECs and glial cells were further analyzed using immunocytochemistry and gene expression analysis.

### 2.4 Quantification of viral genomes

Absolute qPCR was used for quantification of viral genomes (vg) in both the produced AAV‐BR1‐NPC2‐eGFP vector stock, DNA purified from tissues of transduced mice, transduced cells, and from cell culture medium collected 24 h after virus administration *in vitro*. For DNA purification from mBECs, cells from three filter inserts were pooled as one sample, while DNA from glial cells was purified from cells from one well in a 12‐well plate. DNA from the different tissues and cells were purified using AllPrep DNA/RNA Mini Kit (Qiagen, #80204) according to the manufacturer's protocol and quantity and purity were subsequently determined with a DeNovix Spectrophotometer DS‐11. Maxima SYBR Green Master Mix, with ROX as a reference (Thermo Fisher Scientific, #K0223) was used for the qPCR with 0.3 μM forward and reverse primers specific for the CAG promoter region used in the applied AAV2 viral vectors (5′‐AAC GCC AAT AGG GAC TTT C‐3′ and 5′‐GTA GGA AAG TCC CAT AAG GTC A‐3′). All samples were run in triplicates, plasmid DNA encoding the CAG promoter was used to generate the standard curve, and non‐template samples served as negative controls. The qPCR reaction was analyzed using the QuantStudio 6 Flex Real‐Time PCR System (Thermo Fisher Scientific) with an initial denaturation at 95°C for 10 min followed by 40 cycles of denaturation at 95°C for 15 s and annealing/extension at 60°C for 1 min, followed by a melt curve analysis (60–95°C, 0.05°C/s). Viral copy numbers in tissue and cell samples were subsequently normalized to total DNA concentrations.

### 2.5 Relative quantification of NPC2 gene expression

NPC2 gene expression was quantified based on relative RT‐qPCR with hypoxanthine phosphoribosyltransferase 1 (HPRT1) as the reference gene. RNA from the different tissues and cells were purified using AllPrep DNA/RNA Mini Kit according to the manufacturer's protocol and the quantity and purity were subsequently determined with the DeNovix Spectrophotometer DS‐11. For RNA purification from mBECs, cells from three filter inserts were pooled as one sample while RNA from glial cells was purified from cells from one well in a 12‐well plate. Purified RNA was initially treated with RNase‐free DNase I (Thermo Fisher Scientific, #EN0525), according to the manufacturer's protocol, to eliminate the remaining genomic DNA. Shortly, 400 ng or 150 ng RNA purified from tissue or cells, respectively, were incubated with 1 U DNase I in Reaction Buffer diluted in DPEC‐treated water at 37°C for 30 min, and the DNase I enzyme was subsequently inactivated by the addition of EDTA and 10 min incubation at 65°C. cDNA synthesis was then performed on DNase‐treated RNA samples using Maxima H Minus First‐strand cDNA Synthesis Kit (Thermo Fisher Scientific, #K1651), according to the manufacturer's protocol. Briefly, 200 ng (tissue samples) or 100 ng (cell samples) DNase‐treated RNA was used as the template for the cDNA synthesis reaction with 25 pmol random hexamer and oligo (dT)_18_ primers, 0.5 mM dNTP Mix, and Maxima H Minus Enzyme Mix in RT buffer. The reaction was incubated at 25°C for 10 min to ensure primer annealing, polymerization at 50°C for 30 min followed by 5 min of incubation at 85°C in a Veriti™ 96‐Well Thermal Cycler (Applied Biosystems). The RT‐qPCR reaction was then performed as a multiplex probe‐based reaction with TaqMan Fast Advanced Master Mix (Thermo Fisher Scientific, #4444556), FAM conjugated mNPC2 primer‐probe mix (Thermo Fisher Scientific, #4331182, assay ID: Mm00499230_m1) and VIC conjugated mHpRT1 primer‐probe mix (Thermo Fisher Scientific, #4448490, assay ID: Hs02800695_m1), according to the manufacturer's protocol. 2.5 ng (cell samples) or 5 ng (tissue samples) cDNA was used as the template. The reaction was run in a QuantStudio 6 Flex Real‐Time PCR System (Thermo Fischer Scientific) with initial uracil‐N‐glycosylase digestion at 50°C for 2 min, to degrade potential contaminating carryover amplicons, followed by an initial denaturation at 95°C for 2 min, and 40 cycles of denaturation for 1 s at 95°C and annealing/extension for 2 min at 60°C. Relative gene expressions were subsequently calculated from obtained threshold cycle (Ct) values based on the ∆∆Ct method (Livak & Schmittgen, 2001), using non‐transduced control samples as a calibrator.

### 2.6 *In vitro* NPC2 replacement assay

An NPC2 deficient human skin fibroblasts cell line (GM18455) (maximum passage number used P#10), kindly provided by Christian W. Heegaard (Department of Molecular Biology and Genetics – Molecular Nutrition, Aarhus University), was used to evaluate the biological effects of secreted NPC2 (Hede et al., 2020). The applied NPC2 deficient human skin fibroblast cell line (GM18455) holds a nonsense mutation changing the residue Glu‐1 to a stop codon, resulting in a truncated protein at one allele and a missense mutation on the other allele caused by a changed Cys‐28 residue to a Phe resulting in a disrupted di‐sulfate bridge causing a misfolded NPC2 protein. NPC2 deficient fibroblasts are characterized by a massive lysosomal cholesterol accumulation because of an inability of these cells to transport cholesterol from the late‐endosomal lysosomal system caused by the lack of functional NPC2 protein (Hede et al., 2020; Vanier & Millat, 2004). The cell lines used are not listed as a commonly misidentified cell line by the International Cell Line Authentication Committee and no further authentication, besides immunolabeling for cell‐specific markers, were performed in the laboratory. NPC2 deficient fibroblasts were incubated for 48 h with conditioned media from mBECs transduced with the AAV‐BR1‐NPC2‐eGFP vector to evaluate whether secretion of recombinant NPC2 to the media could reverse the cholesterol accumulation. Untreated NPC2 deficient fibroblasts were included as negative controls, and media from non‐transduced mBECs were included as a control for endogenous secretion of NPC2. NPC2 deficient human skin fibroblasts were maintained in DMEM (high glucose) (Life Technology, #31966) supplemented with 10% FCS and 10 μg/ml gentamicin sulfate and seeded in uncoated 24 well plates at a density of 6000 cells/cm^2^. NPC2 deficient fibroblasts treated with conditioned media from AAV‐BR1‐NPC2‐eGFP transduced mBECs and non‐transduced mBECs were used for immunocytochemistry and Filipin III staining.

### 2.7 Immunocytochemistry and Filipin III staining

Immunocytochemistry was performed on mBECs seeded on hanging culture inserts or NPC2 deficient fibroblasts seeded on coverslips. Prior to immunocytochemistry, the cells were washed twice in PBS, fixed in 4% paraformaldehyde for 10 min at room temperature, and washed twice in PBS. All subsequent incubations were performed at room temperature under mild agitation. The fixed cells were blocked for 30 min in a blocking buffer containing 3% bovine serum albumin (Europa Bioproducts, #EQBAH62) and 0.3% Triton‐X‐100 in potassium‐containing phosphate‐buffered saline (PPBS), to avoid unspecific binding. All cells were incubated with primary antibodies diluted in blocking buffer for 1 h. mBECs were incubated with a primary goat anti‐GFP antibody (Abcam, #ab6673) diluted 1:500 combined with either mouse anti‐zonula occludens (ZO1) (Invitrogen, #339100), rabbit anti‐claudin 5 (CLD5) (Merck KGaA, #SAB4502981), or rabbit anti‐NPC2 (Novusbio, #NBP1‐84012) primary antibodies diluted 1:250 in blocking buffer. Fibroblasts were incubated with a primary mouse anti‐vimentin antibody (Agilent, #MO725 clone V9), diluted 1:500 in blocking buffer. To remove unbound antibodies, the cells were subsequently washed in wash buffer (blocking buffer diluted 1:50 in PPBS). The cells were then incubated for 1 h with secondary antibodies diluted 1:250 in blocking buffer. mBECs were incubated with a secondary donkey anti‐goat Alexa 488 (Invitrogen, #A11055) combined with either a goat anti‐mouse Alexa 594 (Invitrogen, #A11032) (for GFP and ZO1 double staining) or a donkey anti‐rabbit 594 (Invitrogen, #A21207) (for GFP and CLD5/NPC2 double staining). Fibroblasts were incubated with donkey anti‐mouse 488 (Invitrogen, #A21202). Following incubation with secondary antibodies, the cells were washed in PPBS and the nuclei of mBECs were counter‐stained using DAPI diluted 1:500 in PPBS for 5 min followed by two washing steps. Fibroblasts were on the other hand stained for 1 h with 10 μg/ml Filipin III diluted in PPBS, from a stock solution of 0.5 mg/mL Filipin III (Merck KGaA, #F4767) diluted in DMSO and subsequently washed with PPBS. Filipin III binds specifically to free cholesterol and can, therefore, be used to assess cholesterol depositions in the cells (Hede et al., 2020). Finally, all cells were mounted using Dako Fluorescent mounting media on object slides. Antibody and Filipin III staining were examined with an AxioObserver Z1 fluorescence microscope equipped with ApoTome and Axiocam MR camera. Acquired Images were subsequently corrected for brightness and contrast using the ImageJ software.

### 2.8 Quantification of filipin III intensity (cholesterol load)

To evaluate changes in the cholesterol load of NPC2 deficient fibroblasts, Filipin III intensities were quantified (Hede et al., 2020). Fibroblasts used for quantification were all stained on the same day with Filipin III and anti‐vimentin antibodies. The vimentin staining alone was used to localize the cells during image acquisition to ensure blinded selection of areas. Filipin III exposure time was fixed for all situations based on untreated NPC2 deficient fibroblasts. Ten to 11 images were acquired for quantification from each situation in the NPC2 replacement assay. Filipin III intensities were measured in ImageJ as the sum of gray values in pixels above a defined threshold. The pixel intensity threshold was defined based on the pixel intensity histogram just after the earliest major peak, to eliminate the low Filipin III intensities caused by cholesterol in cell membranes. To account for varying cell sizes and densities, Filipin III intensities were averaged per cell for each image. Intensities were subsequently normalized as a percentage of the intensity of untreated NPC2 deficient fibroblasts.

### 2.9 Ethical approval

All procedures and handling of mice were approved by the Animal Experiments Inspectorate under the Danish Ministry of Food, Agriculture and Fischeries (license no. 2018‐15‐0201‐01467) and in accordance with the European Legislation of Animal Experimentation 2010/63/EU.

### 2.10 Animals

Twenty female BALB/cJRj mice 7–8 weeks of age (18–22 g), supplied by Janvier Lab (Le Genest‐Saint‐Isle, France), were included in the study. The animals were acclimatized to local environment conditions (20–24°C, 40%–60% humidity, and a 12‐h light/dark cycle) for at least 14 days before the experiment. The mice were housed in standard cages with five animals/cage and all experimental groups were represented in each cage during the study period. The animals were provided with a commercial diet (Altromin 1324, Brogaarden, Gentofte) and water ad libitum. When needed, the animals were anesthetized using isoflurane, which gives a rapid and reliable onset and recovery from anesthesia. Furthermore, the depth of anesthesia is also more readily controlled using isoflurane, compared to other types of anesthetics, which was important during the scanning sessions and euthanasia.

### 2.11 In vivo transduction assay

The mice were block‐randomized into three groups based on body weight. In the first and second groups, the mice were injected intravenously using the tail vein with either AAV‐BR1‐Luciferase (*n* = 6) or AAV‐BR1‐NPC2‐eGFP (*n* = 7) at a dose of 5 × 10^10^ and 1.5 × 10^11^ viral vectors/mouse, respectively. Route of administration, drug doses, and the number of animals were based on a previous study using the AAV‐BR1 vector (Körbelin et al., 2016), and no exclusion criteria were pre‐determined. The third group included PBS‐injected controls (CTRL) (*n* = 7) receiving the same volume of PBS as the mice injected with viral vectors (200 μl) (Figure 4a). All injections were performed in awake animals, in a blinded manner, between 9 and 12 am. The animals were monitored 30 min post‐injection for any signs of hypersensitivity reactions, which were absent for all mice. Bodyweight was monitored once weekly during the entire study period. An experimenter blinded to group status undertook all procedures and post‐staining image analysis. One animal from the AAV‐BR1‐NPC2‐eGFP group was excluded from the study after receiving a lower intravenously viral concentration, resulting in *n* = 6 animals in the AAV‐BR1‐NPC2‐eGFP group, and a total of 19 animals used in the study.

### 2.12 In vivo bioluminescence imaging

To evaluate the transgene expression of luciferase in vivo, AAV‐BR1‐Luciferase mice were scanned at different time points during the study period, however always at the same time of the day (between 9 and 12 am) and compared to the CTRL group (Figure 4a). Before scanning, the mice were injected intraperitoneally with d‐luciferin (150 mg/kg) diluted in PBS (15 mg/ml, XenoLight, PerkinElmer) and anesthetized in an induction chamber using isoflurane (2.5%) in oxygen for 3 min after the injection. The anesthesia was maintained by a nose cone with isoflurane (1%–2%) during the scanning session. Right after the animals were anesthetized, an ophthalmic ointment was applied to both eyes to avoid desiccation. Ten to 20 min after the d‐luciferin administration, bioluminescence imaging was taken in the dorsal and lateral positions (In‐vivo Xtreme II, Bruker). Bioluminescence was detected using the same exposure settings for all mice (120 s). To provide information regarding anatomical localization, an X‐ray image was taken simultaneously with the bioluminescence imaging. The analysis of the bioluminescence signal and its alignment with the X‐ray data were performed using Bruker Molecular Imaging software. When recovering from the anesthesia, the animals were provided with heat using a heat lamp to avoid hypothermia. All animals were allowed to recover fully from the anesthesia, before entering their home cage.

### 2.13 Euthanasia and tissue processing

Eight weeks post‐injection with the viral vectors, the mice were deeply anesthetized with isoflurane (5%), and when reflexes were absent, the chest was opened, and a blood sample was collected intracardially using an EDTA‐flushed syringe (10%, 0.5 M). The blood samples were kept on ice until centrifugation at 2000 × *g* for 10 min at 4°C to collect plasma. Plasma samples were stored at −80°C until further analysis. The animals were euthanized by transcardiac perfusion with 20 ml 0.01 M PPBS (pH 7.4), also known as exsanguination. Brains were dissected into left and right hemispheres and processed for biochemical and immunohistochemical analyses. Organs of interest for biochemical analysis were dissected and snap‐frozen in liquid nitrogen and stored at −80°C. Tissues for immunohistochemical examination were dissected and post‐fixed in 4% paraformaldehyde at 4°C overnight, extensively washed in PPBS, transferred into 30% sucrose solution for a minimum of 48 h, and further processed for cryostat sectioning. The brains were embedded in Tissue‐Tek® O.C.T.™ Compound (Sakura Finetek Europe B.V.) at −21°C and 40 μm serial coronal brain sections were cut on a cryostat (Leica CM3050 S, Germany). The sections were collected free‐floating in PPBS in a sequential series of six. Brain sections were kept in anti‐freeze solution at −20°C until further analysis.

### 2.14 Immunohistochemistry

To block for non‐specific binding of antibodies, the brain sections were incubated in a blocking buffer consisting of 3% porcine serum diluted in 0.01 M PPBS with 0.3% Triton X‐100 (Merck KGaA, #X100) for 30 min at room temperature. The sections were then incubated overnight at 4°C with the following primary antibodies: goat anti‐GFP (Abcam, #ab6673, 1:500), rat anti‐cluster of differentiation molecule 11b (CD11b) (Bio‐Rad, #MCA 711, 1:500), rabbit anti‐neuronal nuclei antigen (NeuN) (Abcam, #ab177487, 1:500), rabbit anti‐glial fibrillary acidic protein (GFAP) (Dako, #Z0334, 1:500), rabbit anti‐glucose transporter 1 (GLUT1) (Abcam, #ab115730, 1:200), and rabbit anti‐NPC2 (Novusbio, #NBP1‐84012, 1:200) diluted in blocking buffer. On the following day, sections were washed in washing buffer (blocking buffer diluted 1:50 in PPBS) and incubated for 30 minutes at room temperature with secondary donkey anti‐goat IgG Alexa 488 (Invitrogen, Thermo Fisher Scientific, #A‐11055), goat anti‐rat Alexa 594 (Invitrogen, #A‐11007), or donkey anti‐rabbit IgG Alexa 594 (Invitrogen, #A‐21207), diluted 1:200 in blocking buffer. Sections stained with NPC2 were added with a secondary peroxidase‐conjugated swine anti‐rabbit IgG antibody (Agilent, #P021702‐2) and visualized using a VECTASTAIN® Elite Avidin‐Biotin Complex (ABC) (VECTOR laboratories, BIONORDIKA DENMARK, #PK6100) followed by the addition of 3,3′‐diaminobenzidine tetrahydrochloride (DAB). All fluorescent NPC2 stains were enhanced with tyramide using the following setup. The tyramide‐enhanced sections were likewise stained using the peroxidase‐conjugated swine anti‐rabbit IgG antibody and added with ABComplex Vector stain for 30 minutes and washed in PPBS. Tyramide Signal Amplification (TSA) Biotin kit (AKOYA Biosciences, #NEL700A001KT) were added for 5 min, the sections washed in PPBS, and incubated with the ABComplex Vectorstain for 30 min. The sections were again washed in PPBS and visualized using an anti‐streptavidin Alexa 594 (Invitrogen, #S32356) antibody for 1 h. After an additional washing step in PPBS, all sections were counterstained with 4′,6‐diamino‐2‐phenylindole (DAPI) (diluted 1:500 in PPBS). Sections were mounted with fluorescent mounting media (DAKO, #S3023), or Pertex (for DAB staining). The fluorescent brain sections were imaged with an Observer Z1 fluorescence microscope equipped with an ApoTome and Axiocam MR camera (Carl Zeiss), while DAB stainings were imaged with an Axioplan 2 microscope equipped with an Axiocam MRc camera (Carl Zeiss). Images were analyzed using the ImageJ software and adjusted for brightness and contrast.

### 2.15 Quantification of NPC2 in plasma and brain homogenates

Brain tissue samples were homogenized in Neuronal Protein Extraction Reagent (N‐PER) (Thermo Fisher Scientific, #87792) supplemented with cOmplete™, Mini, EDTA‐free Protease Inhibitor Cocktail (Merk KGaA, #11836170001) on ice for 10 min and centrifuged at 10,000 × *g* for 10 min at 4°C. The total protein concentrations for brain and plasma samples were determined in duplicates by Pierce™ BCA Protein Assay Kit (Thermo Fisher Scientific, #23225) at 562 nm by using an EnSpire Multimode Plate Reader (Perkin Elmer). The concentrations of mouse NPC2 in plasma and brain homogenates were evaluated using a mouse NPC Intracellular Cholesterol Transporter 2 (NPC2) ELISA kit (Abbexa, #535660) based on the sandwich ELISA technology, according to the manufacturer's protocol. In brief, a seven‐point standard curve was prepared using twofold serial dilutions ranging from 40 to 0.63 ng/ml after which standards, protein samples, and plasma samples were added to a 96‐well plate pre‐coated with primary antibody. The plate was then incubated for 2 h at 37°C. Samples and standards were then aspirated and Detection Reagent A added followed by a 1‐h incubation at 37°C. The Detection Reagent A was then removed, and the plate was washed three times with the included Wash Buffer. Next, Detection Reagent B was added and the plate was incubated for 1 h at 37°C and subsequently washed with Wash Buffer. Finally, tetramethylbenzidine (TMB) substrate was added and incubated for 10 min after which the reaction was stopped by the addition of Stop Solution. Optical density was measured at 450 nm using an EnSpire Multimode Plate Reader (Perkin Elmer). All standards and samples were measured in duplicates. Data were analyzed in GraphPad Prism 9.1.1 using the sigmoidal 4‐parameter logistics curve model to interpolate unknowns based on a standard curve, and the mean absorbance for each set of duplicates was subsequently calculated.

### 2.16 Statistical analysis

**FIGURE 2:**
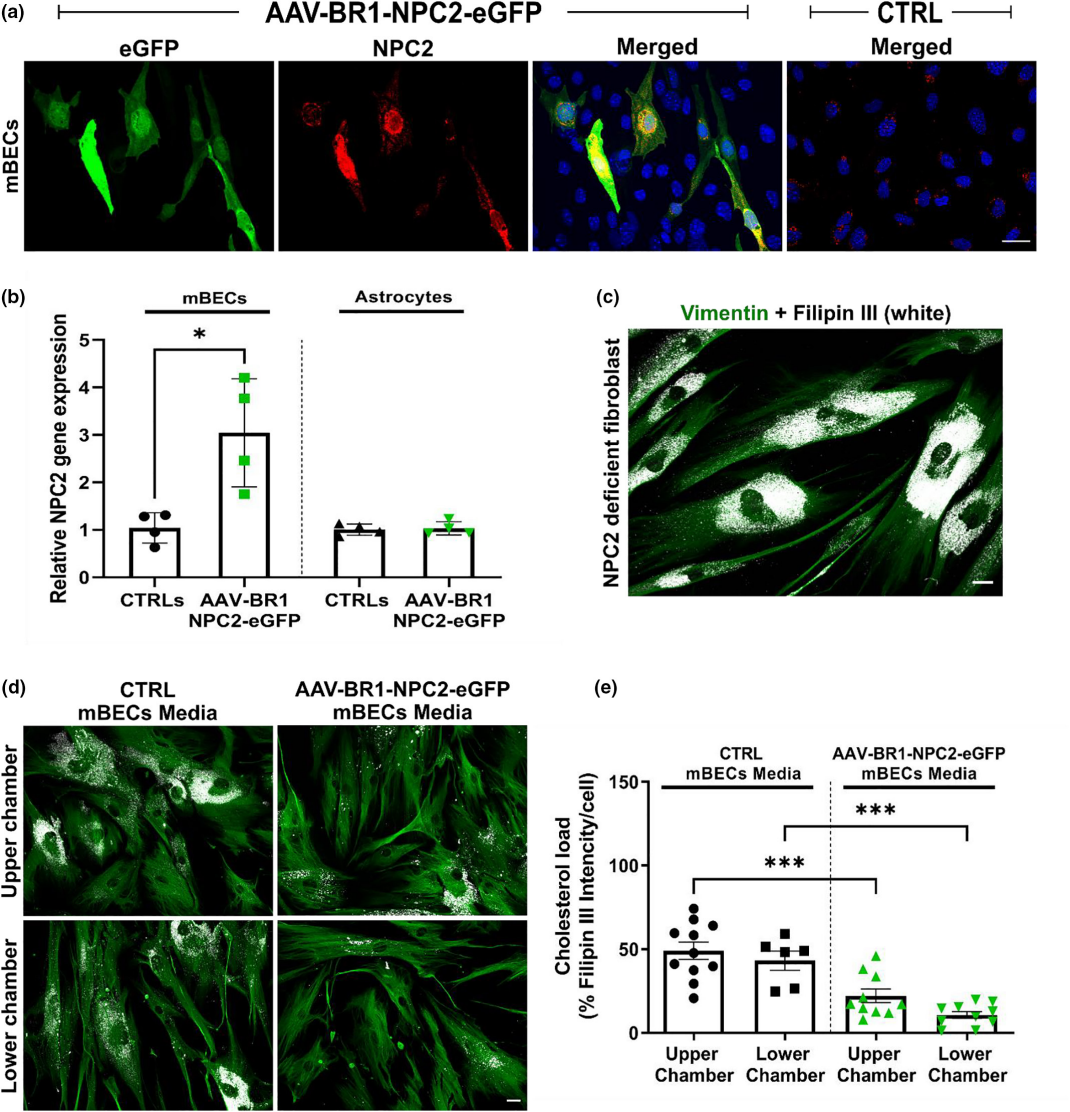
*In vitro* expression of Niemann Pick type C2 (NPC2) protein in mouse brain endothelial cells (mBECs) and primary mice glial cells after transduction with AAV‐BR1‐NPC2‐eGFP and evaluation of the therapeutic effect of the secreted recombinant NPC2 in NPC2 deficient fibroblast. (a) Double labeling of enhanced green fluorescent protein (eGFP) (green) and NPC2 (red) antibodies reveals co‐localization in transduced mBECs, while no eGFP‐positive mBECs are seen among the non‐transduced controls (CTRL). Perinuclear NPC2 expression is observed in all cells including CTRLs reflecting the endogenous expression of NPC2. Nuclei were counterstained with DAPI (blue). Scale bar 25 μm. (b) NPC2 gene expression is significantly increased in transduced mBECs compared to non‐transduced CTRL when analyzing the relative gene expression using RT‐qPCR 48 h after transduction (*t*[6] = 3.39, *p = 0.0147). No significant difference in NPC2 expression is found in glial cells co‐cultured with transduced or CTRL mBECs (*t*[6] = 0.2998, *p* = 0.7745). Data were analyzed using unpaired two‐tailed t‐tests and are presented as mean ± SD (*n* = 4 RNA samples obtained from three inserts for mBECs and one well for glial cells). (c) NPC2 deficient human skin fibroblasts express the fibroblast marker vimentin (green) and are characterized by massive perinuclear cholesterol accumulation corresponding to lysosomes visualized by Filipin III (white). Scale bar 25 μm. (d) Cholesterol accumulation in NPC2 deficient human skin fibroblasts is markedly reduced following treatment with a conditioned medium from either non‐transduced (left panel) or transduced (right panel) mBECs co‐cultured with glial cells. Scale bar 25 μm. (e) Quantification of the cholesterol load in fibroblasts after treatment was evaluated based on Filipin III staining and shows a reduction of 49% (upper chamber) and 43% (lower chamber) of the cholesterol load in untreated fibroblasts following treatment with conditioned medium from non‐transduced CTRL mBECs. Treatment with conditioned medium from transduced mBECs causes a significantly further reduction to 22% (upper chamber) and 11% (lower chamber) demonstrating secretion of functional recombinant NPC2 from transduced mBECs in both the luminal (upper chamber) and abluminal (lower chamber) direction. Data are presented as mean ± SD (*n* = 6–11 images) and analyzed using a one‐way ANOVA (*F*[3,33] = 18.03, *p* < 0.0001) with Tukey's multiple comparisons (***p = 0.0003 for upper vs. upper chamber, and ***p = 0.0002 for lower vs. lower chamber).

**FIGURE 3:**
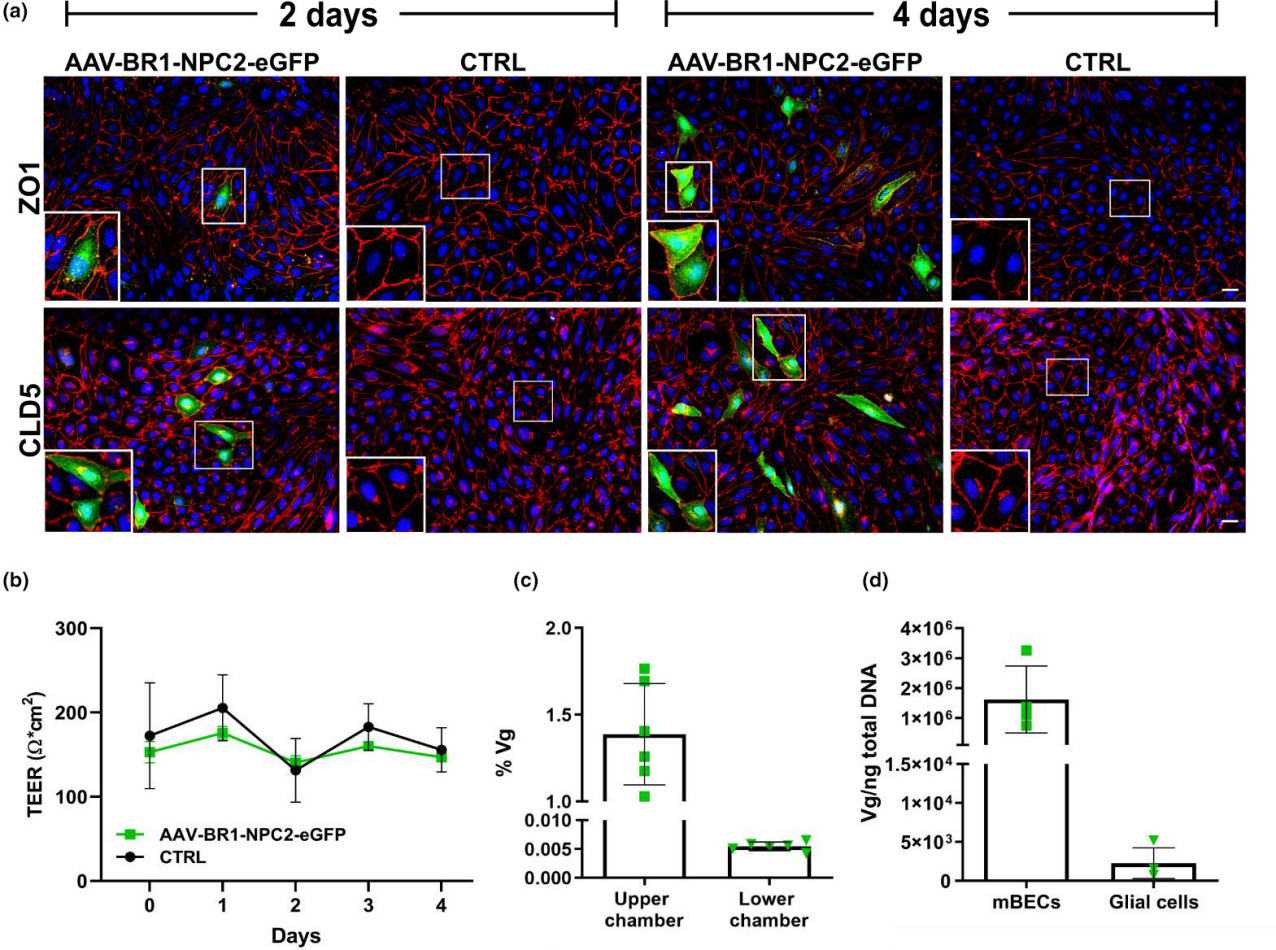
Viral vector distribution of AAV‐BR1‐NPC2‐eGFP and barrier integrity following transduction of an *in vitro* blood–brain barrier (BBB) model using primary mouse brain endothelial cells (mBECs) in non‐contact co‐culture with primary mouse glial cells. (a) The expression of two important tight junction proteins (Zonula occludens 1 (ZO1) and Claudin 5 (CLD5)) was evaluated in the mBECs using immunocytochemistry. Transduced cells expressing enhanced green fluorescent protein (eGFP) (green) are found both 2 and 4 days after virus administration with the majority of positive cells being present after 4 days. In both cases, transduced mBECs maintain a robust tight junction expression (red) similar to that observed in the respective non‐transduced controls (CTRL). Nuclei are counterstained with DAPI (blue). Scale bar 25 μm. (b) Barrier integrity of primary mBECs in non‐contact co‐cultures with primary mouse glial cells, measured as TEER, is not affected by the transduction of mBECs. Data were analyzed using a REML mixed‐effects model with Sidaks multiple comparisons revealing no significant differences between TEER values of transduced mBECs (*n* = 25 culture inserts) and non‐transduced CTRL (*n* = 17 culture inserts). Data are presented as mean ± SD. (c) Viral vectors can cross the *in vitro* BBB model following transduction with 10^10^ viral vectors per culture insert added to the upper chamber, representing the blood side of the BBB. 24 h post‐transduction remaining viral particles in the cell medium (presented as the percentage of viral genomes (vg) in relation to the total number of vg added to the upper chamber) are mainly present in the upper chamber, while only a very small fraction is present in the lower chamber representing the brain side. The majority (98%) of vg is taken up by the cells. Data are presented as mean ± SD (*n* = 6 culture inserts). (d) Viral vectors primarily transduced mBECs in the *in vitro* BBB model when analyzing the presence of viral genomes (vg) within the primary mBECs and glial cells 24 h after vector administration. Data are presented as mean ± SD (*n* = 4 number of DNA samples obtained from three inserts for mBECs and one well for glial cells for data).

**FIGURE 4:**
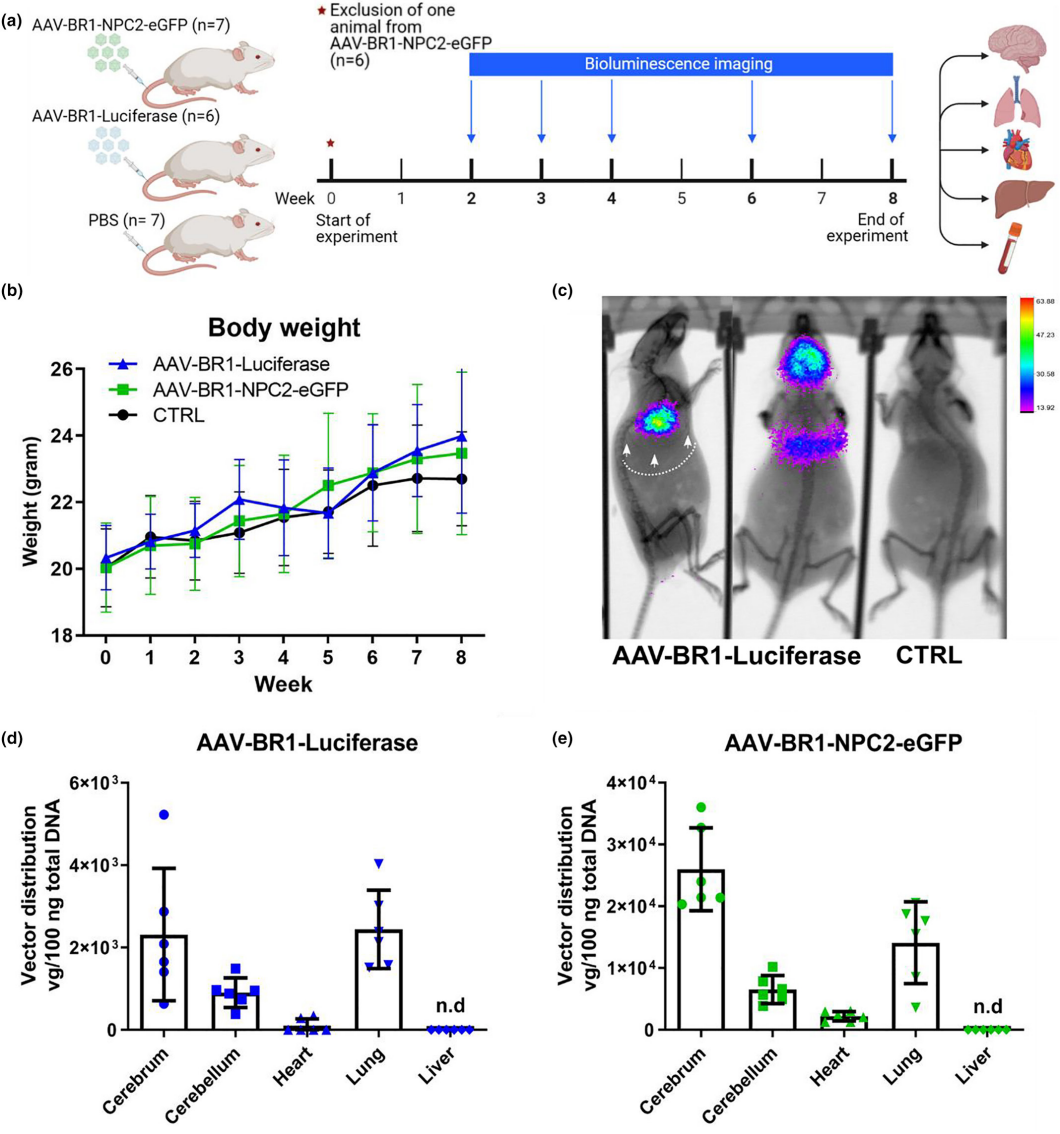
Study design and viral distribution following in vivo transduction. (A) All mice were block‐randomized into three groups according to their body weight and injected in the tail vein with either AAV‐BR1‐Luciferase (*n* = 6 mice), AAV‐BR1‐NPC2‐eGFP (*n* = 6 mice), or saline (PBS) (CTRL) (*n* = 7 mice). By 2–4 weeks post‐injection, AAV‐BR1‐Luciferase mice were scanned weekly for bioluminescence and compared to CTRL mice, and thereafter every second week until completion of the experiments after 8 weeks. At the end of the study, all animals were euthanized and selected organs harvested for further analysis. Created with BioRender.com. (b) Body weight was continuously measured throughout the experiment and analyzed with a two‐way repeated‐measures ANOVA. There were no significant differences in body weight between the three groups (*F*[2,16] *=* 0.1868, *p* = 0.8314). Data are presented as mean ± SD (*n* = 6–7 mice/group). (c) In vivo bioluminescence imaging displaying viral distribution following intravenous injection with the AAV‐BR1‐Luciferase vector or saline (CTRL). Eight weeks after intravenous injection, all mice were positive for luciferase transgene expression in the brain as well as the heart/lung region. Images are representative of all six animals. Images were taken in dorsal and lateral positions and overlayed with an X‐ray image. Arrows point at the lower rib curvature (the dotted line indicates the shape of the rib curvature) to mark the thoracic cavity. (d+e) Biodistribution of AAV‐BR1‐Luciferase and AAV‐BR1‐NPC2‐eGFP viral vectors were analyzed by quantification of viral genomes by qPCR 8 weeks post‐injection. The two vectors showed similar tissue distribution patterns, but with generally higher vectors concentrations in the animals injected with the AAV‐BR1‐NPC2‐eGFP. Both vectors were mainly present in the brain and lung, with low concentration in the heart, while the vectors were non‐detectable (n.d) in the liver. The AAV‐BR1‐NPC2‐eGFP vector showed the highest concentration in the cerebrum compared to the other organs. Data are presented as mean ± SD (*n* = 6 mice/group).

Results are shown as mean with standard deviation (SD). *n* values generally refer to the number of animals for data obtained from in vivo studies while *n* values for data obtained from *in vitro* studies refer to the number of hanging culture inserts for TEER values (Figure 3b) or to the number of DNA or RNA samples obtained from three inserts for mBECs and one well for glial cells for data referring to molecular analysis (Figures 2b and 3d). For data referring to Filipin III intensities, *n* values refer to the number of images analyzed (Figure 2e). No statistical tests were performed to determine sample size, but the number of animals included in the study was based on previous studies of a similar nature using *n* = 3–6 wild‐type animals for vector distribution and protein expression (Davidson et al., 2021; Hughes et al., 2018; Körbelin et al., 2016; Markmann et al., 2018). All statistical analysis was done with a 0.05 significance level using GraphPad Prism (version 9.1.2). All data sets were initially analyzed for normality using the Shapiro–Wilk test and analyzed for outliers using Grubb's test. All data sets passed the Shapiro–Wilk normality test and one data point was excluded from the CTRL group in the data set with relative NPC2 gene expression in cerebellum tissue samples based on Grubb's test. All reported data were tested for equal variances using either an F‐test for datasets containing two groups or a Brown‐Forsythe test for datasets containing more than two groups. In all cases, homogeneity of variance was met, and all data sets were therefore analyzed using parametric unpaired two‐tailed t‐tests (Figures 2b and 7b–d), a parametric one‐way ANOVA with Tukey's multiple comparisons (Figure 2e) or two‐way repeated measures (Figure 4b) ANOVA. TEER values were analyzed using the REML mixed‐effects model with Sidaks multiple comparisons, because of missing values at some time points. Statistical analysis was not performed for data shown in Figures 3c,d and 4d,e.

## 3 RESULTS

### 3.1 Secretion and therapeutic potential of recombinant NPC2 protein

This study investigates the potential of using viral gene therapy to induce the secretion of therapeutic proteins from the BBB using the BEC‐specific AAV‐BR1 viral vector (Körbelin et al., 2016), to increase NPC2 gene expression and protein concentration within the brain. First, we wanted to examine NPC2 expression and secretion from mBECs following AAV‐BR1‐NPC2‐eGFP transduction using an *in vitro* BBB model based on primary mBECs co‐cultured with glial cells (Figure 1c). The vector was designed to induce the production of NPC2 and eGFP as two separate proteins, of which NPC2 was destined for lysosomes within the transduced cells and secretion to adjacent cells, while eGFP was included as a marker for transduced cells because it accumulates within the cells (Figure 1a+b). The AAV‐BR1‐NPC2‐eGFP vector was added to the upper chamber, corresponding to the blood side, for 24 h after which the media containing viral particles was replaced and the cells cultured for an additional 1–4 days. Double labeling the mBECs with eGFP and NPC2 antibodies after 4 days revealed a markedly increased concentration of NPC2 in transduced eGFP‐positive mBECs compared to non‐transduced mBECs (CTRL) (Figure 2a). In AAV‐BR1‐NPC2‐eGFP transduced mBECs, NPC2 was localized perinuclear likely corresponding to a subcellular localization in the lysosomes, while the eGFP staining showed an even distribution of the cytoplasm throughout the cells. Investigations of the relative NPC2 gene expression 48 h after viral transduction revealed a significant (*p* = 0.0147) threefold increase in NPC2 expression in transduced mBECs (3.045 ± 1.14) compared to non‐transduced mBECs (1.043 ± 0.32) (Figure 2b). As shown in the following paragraph (Figure 3), a minor fraction of viral vectors crossed the *in vitro* BBB and entered glial cells, however NPC2 gene expression in the glial cells cultured together with transduced mBECs was not significantly different from glial cells cultured together with non‐transduced mBECs (1.033 ± 0.14 vs. 1.005 ± 0.12) (Figure 2b).

We were unable to analyze the concentration of NPC2 protein being secreted into the cell media because of the lack of a commercially available mouse NPC2 ELISA kit optimized for cell culture media. Instead, we used a previously described NPC2 replacement assay with NPC2 deficient human skin fibroblasts (Hede et al., 2020) to estimate functional NPC2 secretion from mBECs co‐cultured with glial cells following transduction with the AAV‐BR1‐NPC2‐eGFP vector. NPC2 has previously been shown not to cross the BBB both *in vitro* and in vivo (Hede et al., 2020; Nielsen et al., 2011). Therefore, the presence of NPC2 in either the upper or the lower chamber corresponds to the secretion of NPC2 to either of these chambers. Compared to the strong cholesterol accumulation observed in untreated NPC2 deficient fibroblasts (Figure 2c), we observed a pronounced reduction in cholesterol accumulation in NPC2 deficient fibroblasts following the addition of conditioned medium collected from both the luminal and abluminal compartment of mBECs co‐cultured with glial cells (Figure 2d). Quantifications of the cholesterol load in NPC2 deficient fibroblasts revealed a marked reduction after treatment with a conditioned medium from non‐transduced mBEC CTRL to 49.11 ± 17% (upper chamber) and 43.19 ± 14.2% (lower chamber) of the cholesterol load in untreated fibroblasts (100%), indicating secretion of endogenous NPC2 from non‐transduced mBECs (Figure 2e). However, we were able to demonstrate an additional significant reduction in the cholesterol load following treatment with conditioned medium from the mBECs transduced with the AAV‐BR1‐NPC2‐eGFP vector to 22.12 ± 12.7% (upper chamber) and 10.75 ± 6.6% (lower chamber). This indicated that the AAV‐BR1‐NPC2‐eGFP vector facilitated a significantly increased bi‐directional secretion of recombinant NPC2 from mBECs toward both the blood (upper chamber, *p* = 0.0003) and brain (lower chamber, *p* = 0.0002) side of the *in vitro* BBB model compared to non‐transduced cells. The correction of cholesterol in NPC2 deficient fibroblasts cultured with media from transduced mBECs furthermore indicated the secretion of a fully functional recombinant NPC2 protein.

### 3.2 BBB transport of the AAV‐BR1‐NPC2‐eGFP vector

Using the *in vitro* BBB model constructed from primary mBECs co‐cultured with primary mouse glial cells we also investigated the effect of the AAV‐BR1‐NPC2‐eGFP viral vector on the barrier integrity and whether the vector undergoes transport across the BBB. Immunocytochemical detection of two essential tight junction proteins, ZO1 and CLD5 did not indicate any changes in the protein expression either 2 or 4 days after vector administration (Figure 3a). Continuous ZO1 and CLD5 protein expression were observed along the cell–cell borders in both mBECs transduced with AAV‐BR1‐NPC2‐eGFP vector and in non‐transduced CTRL after both 2 and 4 days, even though more eGFP‐positive cells were found after 4 days (Figure 3a). As expected, no eGFP‐positive mBECs were detected among the CTRL mBECs. TEER was measured daily throughout the *in vitro* study as an indicator of barrier integrity and permeability (Thomsen et al., 2021). No significant differences were observed in TEER between the mBECs transduced with AAV‐BR1‐NPC2‐eGFP and non‐transduced CTRLs, at any time point (Figure 3b). Thus, transduction of mBECs co‐cultured with glial cells in the *in vitro* BBB model did not seem to affect the barrier integrity.

To test whether the AAV‐BR1‐NPC2‐eGFP vector was able to cross the *in vitro* BBB model, we collected the medium from the top and bottom chambers, representing the luminal and abluminal side of the BBB, respectively, 24 h after vector administration (10^10^ vg, corresponding to 100%) and examined it for the presence of AAV‐BR1‐NPC2‐eGFP vectors by qPCR. 0.005 ± 0.001% of the total vg (corresponding to 3.6 × 10^2^ ± 50.44 vg/μl) was detected in the bottom chamber, while 1.43 ± 0.29% of the total vg (corresponding to 1.9 × 10^5^ ± 0.4 × 10^5^ vg/μl) remained in the top chamber. Most of the viral vectors added to the top chamber were therefore taken up by the mBCECs. Although the AAV‐BR1‐NPC2‐eGFP vector was also able to cross the *in vitro* BBB model the viral vectors were mainly present in the top chamber 24 hours after virus administration (Figure 3c). Media was likewise collected from non‐transduced cells, but here no viral expression was observed (data not shown). Based on the results regarding the barrier integrity, the observed transport of viral particles across the BBB is not caused by a compromised barrier function.

In addition, we also investigated the vector distribution in the mBECs and glial cells 48 hours post‐transduction using the *in vitro* BBB model and found that the AAV‐BR1‐NPC2‐eGFP vector primarily entered the mBECs (1.6 × 10^6^ ± 1.1 × 10^6^ vg/ng total DNA). Moreover, only very few viral vectors had entered the glial cells (2.2 × 10^3^ ± 1.9 × 10^3^ vg/ng total DNA), despite their ability to cross the mBECs and enter the lower chamber (Figure 3d).

### 3.3 Viral distribution in vivo following intravenous injection.

Then we wanted to examine the distribution and potential of the AAV‐BR1 vector in healthy BALB/c mice, as this viral vector, to our knowledge, never have been investigated in this specific inbred mouse strain. Previous studies with the AAV‐BR1 vector have included FVB/N and C57BL/6 mice (Carroll et al.; 2020; Chen et al., 2020; Dogbevia et al., 2017, 2020; Ivanova et al., 2021; Körbelin et al., 2016; Park et al., 2021; Sundaram et al., 2021). For that purpose, two different constructs were designed and used to study the in vivo distribution of the AAV‐BR1 vector. One construct carrying the luciferase gene under the control of a CAG promoter was used to enable visualization of vector distribution in living animals. The second construct was the same construct used for the *in vitro* studies. Three groups of mice were therefore injected with either AAV‐BR1‐Luciferase (5 × 10^10^ viral vectors/mouse), AAV‐BR1‐NPC2‐eGFP (1.5 × 10^11^ viral vectors/mouse), or PBS (CTRL group), and monitored for 8 weeks followed by euthanasia and organ collection (Figure 4a). During the entire study period, no significant differences were observed in body weight between the three groups and all animals increased their body weight throughout the study (Figure 4b). Furthermore, there were no visible signs of adverse effects after the viral injection in vivo, and immunohistochemical examination for GFAP or CD11b expression did not show any signs of immune reactions from activation of astrocytes and microglia, respectively, in mice injected with AAV‐BR1‐NPC2‐eGFP compared to CTRL animals (Figure S1). Using in vivo bioluminescence imaging, we evaluated the tissue distribution of the AAV‐BR1‐Luciferase vector after intravenous injection. Since previous studies evaluated the bioluminescence signal from approximately 2 weeks post‐injection (Körbelin et al., 2016; Rocha et al., 2011), the first images in this study were planned on post‐injection day 13. The administration of d‐luciferin resulted in detectable bioluminescence signals from the brain in all mice (*n* = 6) from day 13 post‐injection (Figure 4c). Furthermore, all mice displayed varying degrees of luciferase expression in the thoracic cavity. The luciferase signal was evident throughout the entire study period but with increasing intensity over time (data not shown). There was no detectable signal from the liver. Based on the in vivo bioluminescence imaging, the distribution of AAV‐BR1‐Luciferase and AAV‐BR1‐NPC2‐eGFP vector copy numbers was quantified postmortem in brain, heart, and lung tissue using qPCR. Liver samples were also included since previous studies have shown liver genotoxicity after injection with viral vectors (Chandler et al., 2015; Donsante et al., 2001; Nault et al., 2015; Rosas et al., 2012). The distribution of viral vector copy numbers displayed a similar pattern of both the AAV‐BR1‐Luciferase and the AAV‐BR1‐NPC2‐eGFP vectors, hence corresponding to the fact that both vectors contained identical capsid structures and thereby putatively identical targeting mechanisms. Vector concentrations were generally higher in animals injected with the AAV‐BR1‐NPC2‐eGFP vector (10^4^ vg/100 ng total DNA) compared to animals injected with the AAV‐BR1‐Luciferase vector (10^3^vg/100 ng total DNA), corresponding to the higher concentration of viral vectors administered to the AAV‐BR1‐NPC2‐eGFP group compared to the AAV‐BR1‐Luciferase group. In both groups, the viral vectors accumulated primarily in the brain and lung, whereas a minor part of the vectors were distributed to the heart after systemic administration, corresponding well with the bioluminescent images (Figure 4d,e). It was not possible to detect viral vectors in the liver of mice injected with either the AAV‐BR1‐NPC2‐eGFP or AAV‐BR1‐Luciferase (Figure 4d,e). Expectedly, the viral vectors were non‐detectable in organs from CTRL animals (data not shown).

### 3.4 AAV‐BR1‐NPC2‐eGFP brain‐specific transduction

**FIGURE 5:**
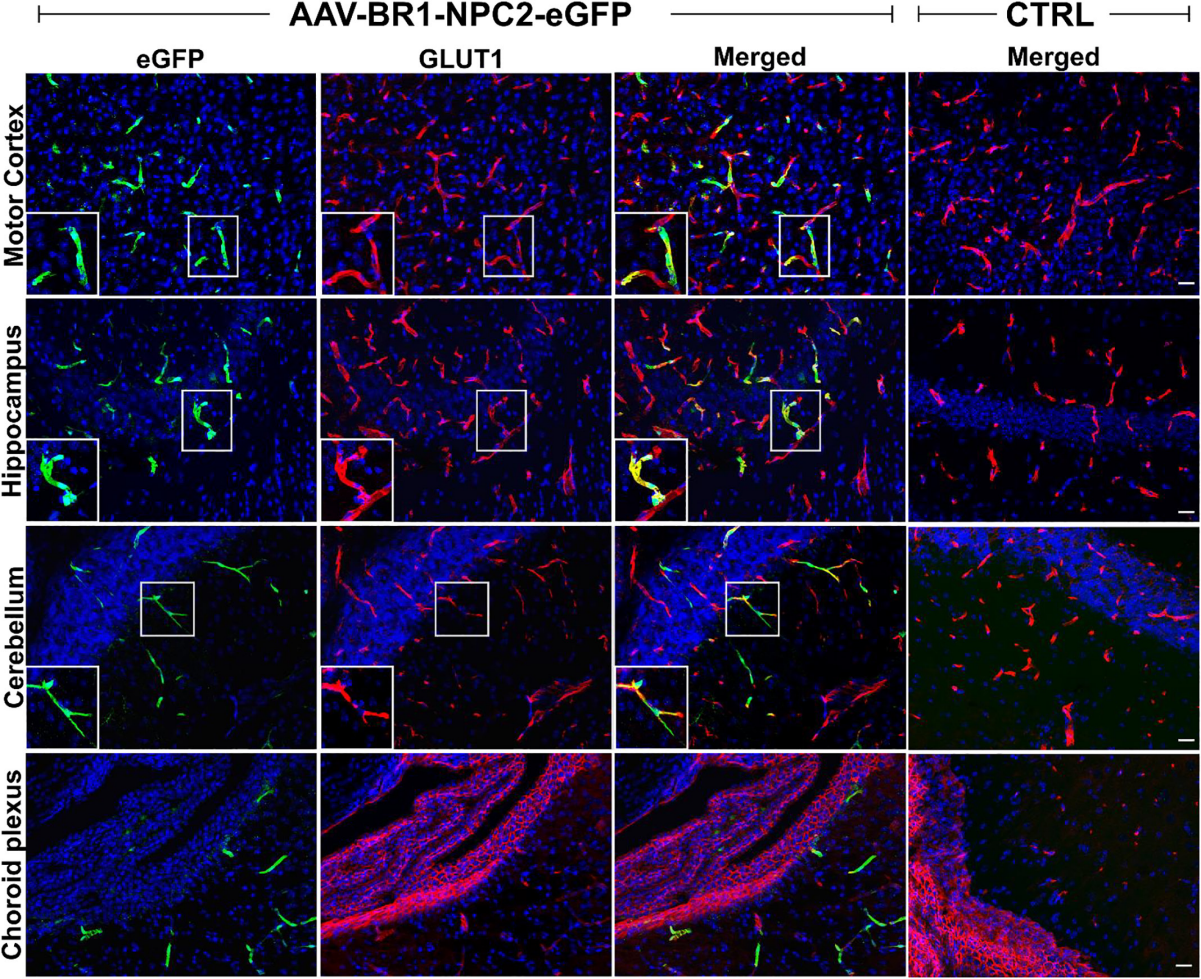
In vivo capillary transduction 8 weeks post AAV‐BR1‐NPC2‐eGFP injection in healthy BALB/c mice. Widespread expression of enhanced green fluorescent protein (eGFP) (green) following transduction is seen in glucose transporter 1 (GLUT1) (red) positive capillaries in different brain regions of the transduced mice. Transductions are brain endothelial cell‐specific, and no eGFP expression is observed in GLUT1‐positive choroid plexus epithelial cells or the capillaries of the choroid plexus (lower row). No eGFP‐expressing cells are observed in the mice injected with saline [control mice (CTRL)]. White boxes show transduced capillaries at higher magnification, to better visualize the co‐localization between eGFP and GLUT1. The images are representative of CTRL (*n* = 7 mice) or AAV‐BR1‐NPC2‐eGFP (*n* = 6 mice). Nuclei are counterstained with DAPI (blue). Scale bar 25 μm.

Next, we wanted to evaluate the AAV‐BR1‐NPC2‐eGFP mediated transduction of brain capillaries, which was our primary target for gene therapy. Immunolabeling for the BEC hallmark protein GLUT1 and the marker for transduction and transgene expression eGFP showed positively transduced capillaries distributed throughout the entire brain in all mice injected with AAV‐BR1‐NPC2‐eGFP (Figure 5). As expected, no eGFP‐positive capillaries were found in the CTRL animals. eGFP fluorescence was not observed in the epithelial cells or capillaries of the choroid plexus (Figure 5, lower panel). Capillaries or neurons (see next paragraph) within the periventricular regions were not labeled more frequently than in other brain regions, indicating no transport of the AAV‐BR1‐NPC2‐eGFP virus across the blood‐cerebrospinal fluid barrier (BCSFB).

**FIGURE 6:**
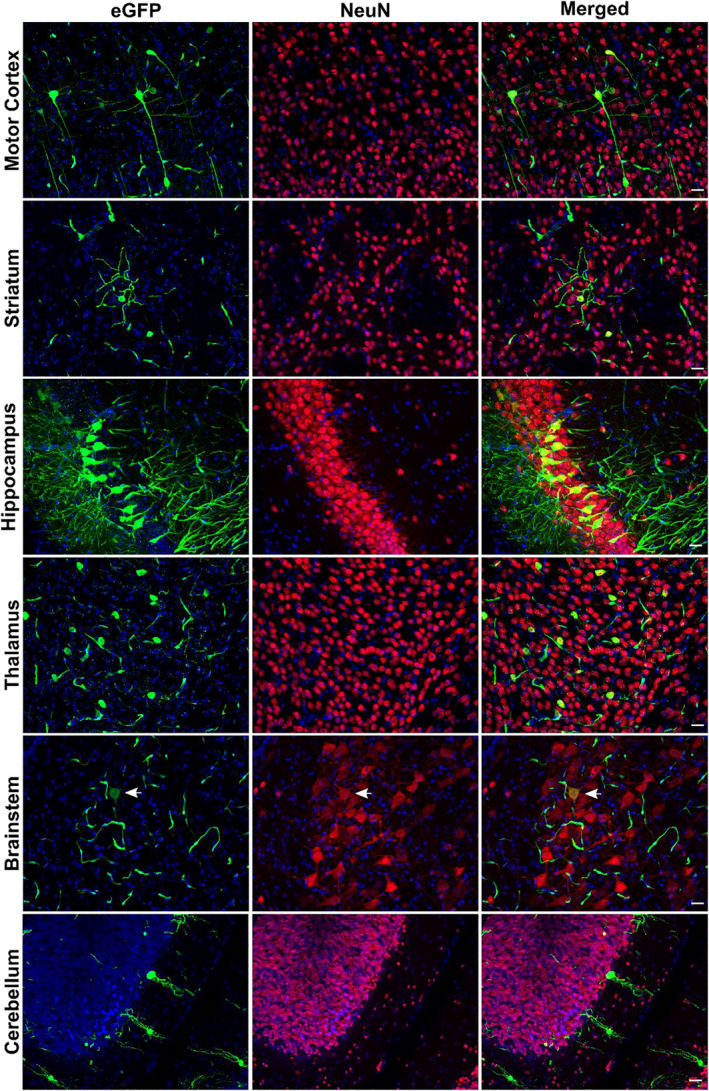
In vivo neuronal transduction 8 weeks after AAV‐BR1‐NPC2‐eGFP injections in healthy BALB/c mice. Expression of enhanced green fluorescent protein (eGFP) (green) following transduction of neuronal nuclei antigen (NeuN)‐positive neurons (red) is observed in several brain regions with particular prominent expression in the hippocampal region (CA3). The images are representative of AAV‐BR1‐NPC2‐eGFP (*n* = 6 mice). In the picture of the brainstem, the arrows identify a transduced neuron. Nuclei are counterstained with DAPI (blue). Scale bar 25 μm.

We also detected cells with morphology corresponding to neurons expressing eGFP in several different brain regions of mice injected with the AAV‐BR1‐NPC2‐eGFP, indicating that the AAV‐BR1‐NPC2‐eGFP vector was able to not only target BECs within the BBB but also to cross the BBB and transduce neurons. Confirmed by immunohistochemistry, the neuronal cell marker NeuN co‐localized with eGFP, revealing that eGFP positivity was because of transduced neurons distributed widely in different regions of the brain with the most prominent occurrence seen in the cerebral motor cortex, striatum, hippocampus (CA3 region), thalamus, and cerebellum (Table 1 and Figure 6). The transduced neurons included pyramidal cells of the hippocampal cortex, Purkinje cells, and basket‐, or stellate cells in the cerebellum, indicating a broad neuronal tropism. This was consistent for all vector‐injected mice, although some brain areas such as pons and medulla oblongata revealed some variety ranging from a few transduced neurons to complete absence (Table 1). It was not possible to identify eGFP‐positive neuronal staining in the hypothalamus or mesencephalon although the fluorescence of eGFP in brain capillaries in these regions was similar to the rest of the brain areas. Immunoreactivity was absent from cells with morphology corresponding to major glial cell types like astrocytes, oligodendrocytes, or microglia (data not shown), thus the viral vectors predominantly occurred intraneuronal when crossing the BBB, and these results are therefore supporting our observation *in vitro*.

**TABLE 1:** Schematic overview of the neuronal distribution of AAV‐BR1‐NPC2‐eGFP in various regions of the mouse central nervous system

Area/Animal	Forebrain					Brainstem			Cerebellum	
	Cerebral motor cortex	Striatum	Thalamus	Hippocampus	Hypothalamic area	Mesencephalon	Pons	Medulla oblongata	Purkinje cells	Basket or stellate cells
1	+	+	+	+	—	—	(+)	—	—	+
2	+	+	+	+	—	—	(+)	—	(+)	+
3	+	+	+	+	—	—	(+)	—	—	+
4	+	+	+	+	—	—	(+)	(+)	(+)	+
5	+	+	+	+	—	—	(+)	(+)	(+)	+
6	+	+	+	+	—	—	–	—	(+)	+

Abbreviations: + positive neuronal staining, (+) weak staining, and/or very few positive neurons. – no neuronal staining.

### 3.5 Recombinant NPC2 expression in the brain following AAV‐BR1‐NPC2‐eGFP transduction

Finally, we wanted to examine whether the AAV‐BR1‐NPC2‐eGFP mediated transductions in the brain lead to increased NPC2 gene expression and protein concentrations similar to what was observed *in vitro*. As NPC2 is an endogenous protein normally found in several cell types (Naureckiene et al., 2000; Ong et al., 2004; Storch & Xu, 2009; Vanier & Millat, 2004), we initially performed a characterization of the normal NPC2 protein levels within the brain of healthy mice based on NPC2 DAB immunohistochemistry (Figure S2). At brain barrier sites, NPC2 was expressed in BECs of the lower brainstem and lightly in choroid plexus epithelial cells. Concerning the labeling of BECs, a consistent finding was graduation in the labeling intensity ranging from virtually absent in the forebrain, over average in diencephalon and mesencephalon to being more prominent in the lower brainstem and cerebellum. Adjoining the varying labeling of BECs, a consistent finding was the prominent labeling of oligodendrocyte somata in both gray and white matter throughout the brain. In the cerebellum, Bergman glia also expressed NPC2. Apart from a blunt appearance of neuronal NPC2 in the hippocampus, a general finding was the lack of NPC2 in neurons, which also included Purkinje cells of the cerebellar cortex (Figure S2).

**FIGURE 7:**
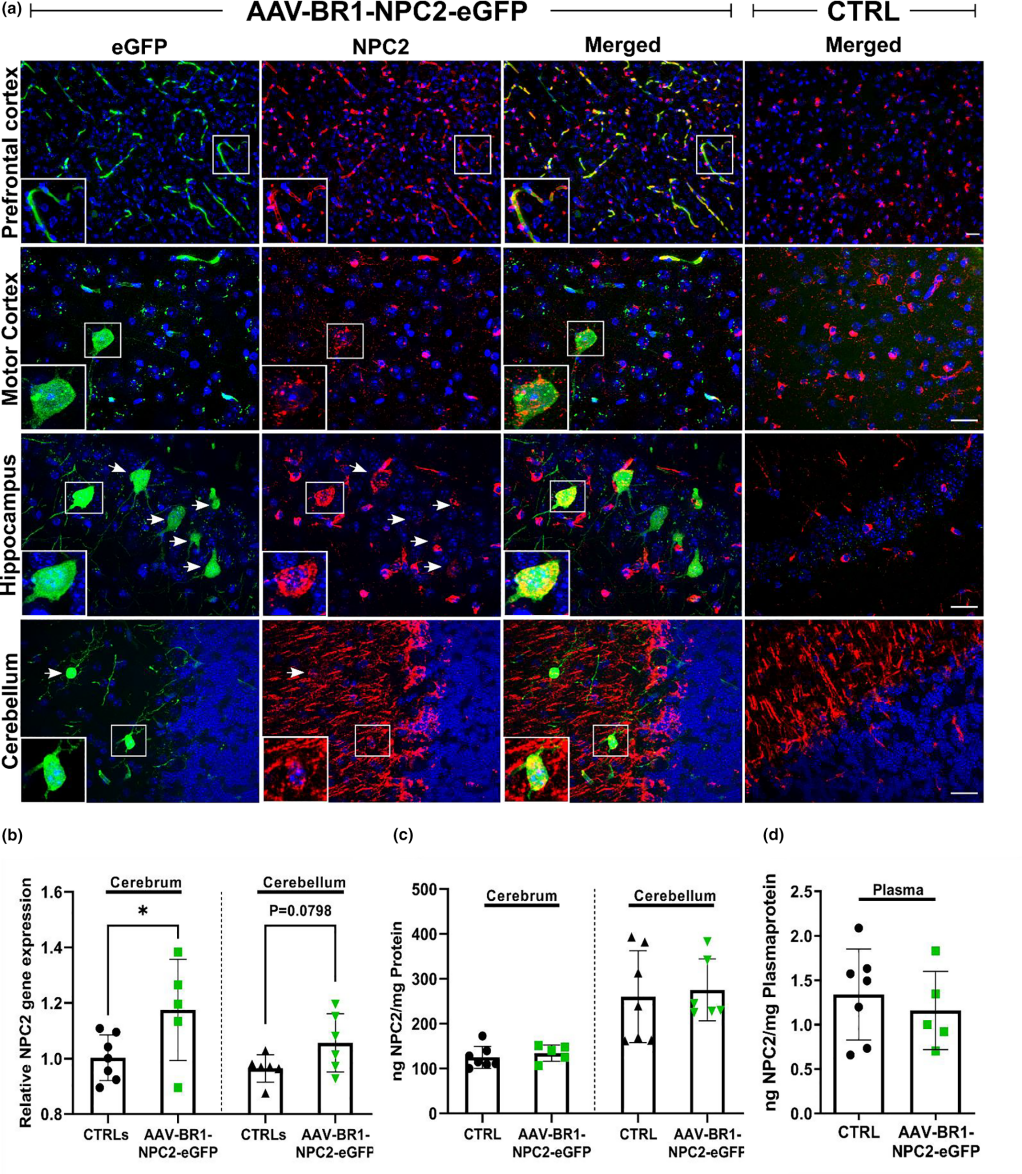
In vivo Niemann Pick type C2 (NPC2) protein expression following transduction with AAV‐BR1‐NPC2‐eGFP in healthy BALB/c mice. (a) NPC2 expression (red) is observed widespread throughout the brain in both transduced and control mice injected with saline (CTRL), as it is an endogenous protein. However, a low or absent NPC2 expression is normally found in the prefrontal cortex capillaries and neurons, respectively. Double labeling with eGFP (green) and NPC2 antibodies reveals co‐localization, and a high recombinant NPC2 expression in the prefrontal cortex capillaries and transduced neurons hence demonstrating de novo NPC2 expression, while no NPC2 expression is observed in prefrontal cortex capillaries or neurons in the saline‐injected CTRL mice. NPC2 expression is, however, widely seen in cells of morphology like oligodendrocytes in both transduced and saline‐injected CTRL mice. Arrows point to NPC2 expression in the transduced neurons in the hippocampus and cerebellum, not highlighted by the higher magnification, shown in the white boxes. The images are representative of AAV‐BR1‐NPC2‐eGFP (*n* = 6 mice) or CTRL (*n* = 7 mice). Nuclei are counterstained with DAPI (blue). Scale bar 25 μm. (b) Analyzing the relative gene expression using RT‐qPCR, NPC2 gene expression is significantly increased in the cerebrum of transduced mice (*n* = 5) compared to saline‐injected CTRL mice (*n* = 7) (*t*[10] = 2.229, *p = 0.0499). In cerebellar samples, only a tendency toward significant increase could be observed in the transduced mice compared to saline‐injected CTRL (*t*[10] = 1.950, *p* = 0.0798. Relative gene expression was compared using an unpaired *t*‐test, and data are presented as mean ± SD. (c + d) No significant differences are observed concerning NPC2 protein concentration in the cerebrum (*t*[10] *=* 0.7046, *p* = 0.4972), cerebellum (*t*[11] = 0.3027, *p* = 0.7678), and plasma (*t*[10] = 0.6329, *p* = 0.541) samples from transduced mice when compared to saline‐injected CTRL mice. NPC2 protein concentrations were measured using a mouse NPC2 ELISA and normalized to total protein concentrations determined using a BCA protein assay. Statistical comparisons are based on unpaired two‐tailed t‐tests and data presented as mean ± SD (*n* = 5–7 mice/group).

As the distribution of NPC2 in BECs was heterogeneous and almost absent in the forebrain in healthy mice, we examined the prefrontal cortex for recombinant NPC2 protein expression mediated by the AAV‐BR1‐NPC2‐eGFP vector. In the prefrontal cortex, intense immunolabeling of recombinant NPC2 was detected in eGFP expressing BECs, while no NPC2 staining in BECs was found in the same area of CTRL animals (Figure 7a, upper panel). Moreover, NPC2 staining was also observed in transduced neurons expressing eGFP, while no NPC2‐positive neurons were observed in the CTRL animals (Figure 7a), hence signifying a recombinant NPC2 protein expression in neurons following intravenous administration of the AAV‐BR1‐NPC2‐eGFP vector. In addition to the increased recombinant NPC2 protein expression observed by immunohistochemistry, NPC2 gene expression was also significantly increased (*p* = 0.0499) in the cerebrum following injections with the AAV‐BR1‐NPC2‐eGFP vector (1.175 ± 0.18 vs. 1.003 ± 0.08 in CTRL) whereas only a non‐significant increase (*p* = 0.0798) in NPC2 expression was observed in the cerebellum (1.056 ± 0.10 vs. 0.9643 ± 0.05 in CTRL) (Figure 7b). It was not possible to demonstrate a significant increased NPC2 protein concentration following injection with the AAV‐BR1‐NPC2‐eGFP vector when analyzing brain homogenates from the cerebrum (134.3 ± 18.34 vs. 125.1 ± 24.83 ng NPC2/mg protein in CTRL) and cerebellum (275.2 ± 68.96 vs. 260.3 ± 102.6 ng NPC2/mg protein in CTRL) (Figure 7c), or in plasma samples (1.160 ± 0.44 vs. 1.339 ± 0.51 ng NPC2/mg plasma protein in CTRL) (Figure 7d) using an mNPC2 ELISA kit.

## 4 DISCUSSION

Here, we prove evidence of viral transduction of primary BECs leading to secretion of fully functional NPC2 protein resulting in therapeutic efficacy with a reduction in cholesterol accumulation in NPC2 deficient fibroblasts. Furthermore, we demonstrate that intravenous injection of the BEC‐specific AAV‐BR1 viral vector in healthy BALB/c mice leads to widespread genetic modification of brain capillaries with the synthesis of recombinant eGFP and NPC2 proteins.

### 4.1 Expression and secretion of recombinant NPC2

We observed increased NPC2 gene expression both *in vitro* and in vivo in the brain following transduction with the AAV‐BR1 viral vector, as well as increased expression and secretion of recombinant NPC2 protein *in vitro*. We previously demonstrated increased NPC2 secretion by BECs following non‐viral gene therapy *in vitro* (Hede et al., 2020). However, this strategy is at risk of leading to the NPC2 secretion being too low to exert a biologic effect, for example, in NPC2 deficient fibroblasts (Hede et al., 2020), as opposed to the results of the present study. Using the AAV‐BR1 viral vector, we are now able to show a therapeutic effect of NPC2 secreted from transduced mBECs *in vitro*, underlining that transduction with the AAV‐BR1‐NPC2‐eGFP vector leads to production and secretion of functional recombinant NPC2 proteins. Some discrepancy remains regarding the interpretation of the main direction of secretion by genetically modified BECs. We previously demonstrated bi‐directional secretion of recombinant NPC2 and erythropoietin *in vitro* with the main secretion being toward the upper chamber, representing the blood side, using an *in vitro* BBB model based on primary rat cells (Burkhart et al., 2017; Hede et al., 2020). The present study demonstrates approximately equal secretion toward the blood (upper chamber) and brain side (lower chamber) using an *in vitro* mouse BBB model. Even though *in vitro* BBB models based on primary cells are assumed to mimic the in vivo situation closely, it is uncertain if these *in vitro* findings regarding the bi‐directional secretion can be translated to the in vivo situation. Unfortunately, we were unable to detect a difference between NPC2 protein concentrations in plasma samples from AAV‐BR1‐injected mice and CTRL. It can therefore be hypothesized that recombinant NPC2 proteins are degraded or cleared from the blood over time leading to normalization of the NPC2 plasma concentration.

Despite clear differences in the expression of eGFP and NPC2 protein between virus‐injected mice and CTRL when examined using immunohistochemistry, it was not possible to detect a significantly increased NPC2 protein concentration in brain homogenates of mice transduced with the AAV‐BR1‐NPC2‐eGFP vector. As we were unable to report a significant increase in the NPC2 concentration in healthy mice, one could speculate that the sample size was insufficient. However, in our opinion, we cannot justify including more animals in the study because of the small effect size (Cohen's *d* = 0.42), resulting in a calculated sample size of 90 mice/group (power of 80% and *α* = 0.05) (Table S1). Even though we were unable to detect a significant difference in the NPC2 concentration, transduction with the AAV‐BR1 vector resulted in an increase of 7.4% and 5.7% in the NPC2 concentration in the cerebrum and cerebellum, respectively. It was previously stated that less than 10% of normal protein levels seems sufficient to exert a therapeutic effect in LSD (Moullier et al., 1993; Sands & Davidson, 2006), and therefore even a small increase in the NPC2 concentration might have beneficial effects in patients suffering from NPC2 deficiency.

### 4.2 AAV‐BR1 gene therapy as a strategy for drug delivery to the brain

Several studies exploring AAV viral‐based gene therapy in the brain rely on intracerebral injection attempting to ensure delivery to the brain with the avoidance of peripheral off‐target effects (Bey et al., 2017; Haurigot et al., 2013; Hughes et al., 2018; Markmann et al., 2018). Targeting the BBB based on intravenous administration is considered especially advantageous in NPC2 and other LSDs affecting young children, as it is a less invasive procedure. Another factor favoring systemic injection is the widespread distribution of the AAV‐BR1 viral vector in the brain achieved by transduction of the BBB, which is considered highly advantageous compared to direct injection into the brain, where the brain distribution may be limited to areas close to the injection site (Pardridge, 2011, 2016). To date, most gene therapy studies have attempted to treat NPC1 deficient mice using AAV9 vectors (Chandler et al., 2017; Hughes et al., 2018; Xie et al., 2017), while only a single study involves NPC2 deficient mice using intracisternal administration of AAVrh.10 vectors (Markmann et al., 2018). If administered systemically the AAV9 vector can cross the BBB and primarily transduce neurons, thereby enabling transgene expression both inside and outside the central nervous system (CNS) (Chandler et al., 2017; Xie et al., 2017). Here, we took a different approach, and to our knowledge this is the first attempt where viral transduction of BECs leads to the production of the NPC2 proteins, possibly enabling secretion both widespread into the brain and the bloodstream. Treating NPC1 deficient mice with the AAV‐BR1 vector would entail a high transduction efficiency because of the transmembrane nature of the NPC1 protein, while the soluble nature of the NPC2 protein makes it more potent because of the possibility of cross correction between genetically modified cells and enzyme deficient cells (Hede et al., 2020; Markmann et al., 2018; Sands & Davidson, 2006). Specifically targeting the BECs at the border between the CNS and the peripheral tissues after intravenous injection with the AAV‐BR1 vector further enhance this potential, because of the possible bi‐directional secretion from the BECs toward both the CNS and the periphery. This makes the AAV‐BR1 a highly promising vector for the treatment of NPC2 disease and other LSD with CNS involvement caused by a lack of secretory protein, for example, Krabbe disease, Gaucher disease, and Mucopolysaccharidoses (Edelmann & Maegawa, 2020).

The additional transduction of neurons and lung tissue observed in this study will probably only improve the treatment efficiency by increasing the NPC2 concentration in the brain and blood, respectively. To limit the transgene expression to endothelial cells of the brain, the specificity of the vector could be further improved by using an endothelial cell‐specific promoter, like the regulatory sequences C‐*Ocln*‐W and C‐*Sls2a1*‐S‐W, which in mice have been shown to enable a stronger and more brain endothelial‐specific expression than the CAG promoter (Graßhoff et al., 2021) used in the present study. Secretion of proteins to the blood by BECs might however have limitations in other settings where the resulting protein shows off‐target effects. This may be the case for some growth factors known to exert neuroprotective effects inside the brain, but cause malignancy when administered systemically (Chang et al., 2019; Cocco et al., 2018; Griffin et al., 2018). The justification for using the strategy where proteins are secreted from BECs is made in conditions with a universal need for therapeutic proteins both systemic and in the brain compromised by the restraints of the BBB. Another interesting approach, apart from attempting to enable protein secretion, could be to modify the endogenous expression of BECs proteins, for example, nutrient transporters, which may have relevance to aid nutrient supply to the pathological brain (Erickson & Banks, 2013; Garwood et al., 2017; Mezzaroba et al., 2019; Szablewski, 2017). In such cases, it will be crucial to consider the observed transduction of neurons as their genetic modification may challenge proper neuronal functioning.

### 4.3 The significance of AAV‐BR1 crossing the BBB

In addition to the desired transduction of BECs, we noticed the capability of the AAV‐BR1 vector to cross the BBB both *in vitro* and in vivo and an ability to transduce neurons in vivo. The same phenomenon has previously been reported upon intravenous administration of the AAV‐BR1 vector, albeit not to the same extent (Dogbevia et al., 2020; Körbelin et al., 2016; Sundaram et al., 2021). Despite the obvious transduction of neurons observed in the current study, BECs remained the majority of cells being transduced by the AAV‐BR1 vector. These findings correlate well with our results *in vitro*, where approximately 98% of the viral vectors were taken up by the mBECs, and only 0.005% of the viral vectors were able to cross the *in vitro* BBB. The mechanism for passaging of the AAV‐BR1 vector through BECs remains unknown except for its unequivocal specific binding to the luminal side followed by further uptake and transport. Wild‐type AAV2 vectors are known to partially cross the BBB and were suggested to cross the BBB through passive diffusion as transendothelial transport was increased in cultured endothelial cells following cold‐induced destabilization of TJ proteins (Merkel et al., 2016). Counteracting this observation, we did not observe any signs of decreased BBB integrity *in vitro* following transduction with the AAV‐BR1‐NPC2‐eGFP vector with regards to TEER or expression of the tight junction proteins ZO1 and CLD5. Supporting our observations, the BBB permeability measured by albumin and IgG extravasation was lowered following intravenous administration of AAV‐BR1 in incontinentia pigmenti, which is a genetic disease characterized by BBB disruption, hence counteracting BBB disruption caused by the administration of AAV‐BR1 vectors (Dogbevia et al., 2017; Körbelin et al., 2016). Theoretically, the viral vectors could also have entered the brain parenchyma through passive diffusion across the BCSFB. A major BCSFB transport, however, seems unlikely, because of the lack of transduced choroid plexus epithelial cells, capillaries within the choroid plexus, or nearby neurons, in contrast to that reported previously with other viral vectors (Chen et al., 2020). Also of note, passive diffusion within the extracellular space of the CNS is very limited (Wolak & Thorne, 2013), making it unlikely that the widespread transduction of several neuronal cell types throughout the brain, especially the cerebral motor cortex, would be achieved through passive diffusion from the choroid plexus.

### 4.4 Extracerebral distribution and safety profile of the AAV‐BR1 vector in BALB/c mice

We demonstrate that the AAV‐BR1 vector very specifically targets the brain and lungs. Previous studies using the identical AAV‐BR1 capsid revealed similar specificity toward the brain (Dogbevia et al., 2017, 2020; Körbelin et al., 2016), although these studies did not observe the same extent of transduction of lung tissue as reported here. Körbelin and colleagues (Körbelin et al., 2016) examined AAV‐BR1 vector distribution in FVB/N mice also used for the development of the AAV‐BR1 vector through in vivo screening of a random AAV2 peptide library. In opposite to our results, analysis of vector distribution by bioluminescent imaging did not reveal any signal from the thoracic cavity even though the same dose of virus particles was administered (5 × 10^10^ vg/mouse) (Körbelin et al., 2016). The AAV‐BR1 vector was also used for gene delivery in a mouse model of Sandhoff disease (Dogbevia et al., 2017, 2020; Körbelin et al., 2016) and incontinentia pigmenti (Dogbevia et al., 2017, 2020; Körbelin et al., 2016), two mouse models both based on the C57BL/6 mouse strain. Both studies report cerebral vector concentrations of approximately 10^5^ vg/100 ng total DNA after injection of 1.8 × 10^11^ vg/mouse, which is higher than observed in the present study, while the reported vector concentration in lung tissue was approximately 10^4^ vg/100 ng total DNA in the Sandhoff's disease mouse model (Dogbevia et al., 2020). The increased transduction of lung tissue, compared to the brain, observed in the present study could, therefore, be related to the use of different mouse strains with the present study using BALB/cJRj mice, instead of C57BL/6 mice. The difference in mouse strain could also contribute to the higher degree of neuronal transduction seen in the present study compared to that previously reported (Dogbevia et al., 2020; Körbelin et al., 2016). Variation in tissue tropism when comparing different mouse strains has previously been reported in a study using another AAV vector serotype, AAV‐PHP.B. Following intravenous injection with the viral vector, efficient CNS transduction was observed in different inbred strains including the C57BL/6N and the FVB/N mice, but the vector was not able to transduce brains of BALB/c mice (Matsuzaki et al., 2019). Thus, it cannot be precluded that these differences regarding lung and neuronal tropism seen with the AAV‐BR1 vector are caused by mouse strain differences. These findings support the need for this thorough characterization of the AAV‐BR1 viral vector in the BALB/c mouse strain investigated in the present study. Furthermore, the NPC2 deficient mouse model is maintained on a BALB/c background, and the results in this study are therefore highly valuable for planning future experiments in this particular disease mouse model.

The transduction of lung tissue does not seem to cause any side effects, as we did not observe any changes in weight or general well‐being of the animals receiving the AAV‐BR1 vector, consistent with previous reports (Dogbevia et al., 2017, 2020; Körbelin et al., 2016). The results of the above‐mentioned studies using the AAV‐BR1 vector and the current study, show very little to complete absence of hepatic involvement. This is favorable when using AAV gene therapy since increased risks of developing hepatocellular carcinoma following accumulation of AAV vectors have previously been reported (Chandler et al., 2015; Donsante et al., 2001; Nault et al., 2015; Rosas et al., 2012). By comparison, AAV9 vectors or peptide‐conjugated AAV vectors reportedly with natural tropism for the CNS (Duque et al., 2009; Gray et al., 2011; Meng et al., 2021; Yang et al., 2014) are often associated with marked vector accumulation and off‐target effects in non‐neuronal tissues, including the liver. The liver, kidney, lung, heart, and spleen of mice injected with the AAV‐BR1 vector revealed no signs of fibrosis or malignancy (Dogbevia et al., 2017).

In conclusion, we demonstrate bi‐directional secretion from *in vitro* transduction of mBECs sufficient to reverse pathological cholesterol accumulation in NPC2 deficient fibroblasts. Furthermore, we demonstrate that BECs can be transduced following intravenous administration of AAV‐BR1‐based gene therapy to induce the production and possibly secretion of recombinant NPC2 protein from the BBB. We achieve high viral concentration in the brain capillaries with limited off‐target distribution, including absence in liver tissue. It remains to further employ this strategy in NPC2 deficient mice where the therapeutic potential of recombinant NPC2 secreted from brain capillaries may reveal both neurological and non‐neurological improvements.

## AUTHOR CONTRIBUTIONS

AB and TM: Conceptualization; CLMR, EH, AB, and TM: Funding acquisition; CLMR, EH, LJR, JK, SSH, and AB: Investigation; JK and AB: Methodology; LBT, AB, and TM: Project administration; JK and MS: Resources; JK, LBT, MS, AB, and TM: Supervision; CLMR, EH, LJR, JK, SSH, and AB: Validation; CLMR, EH, LJR, AB, and TM: Visualization; CLMR, EH, AB, and TM: Writing—original draft; CLMR, EH, LJR, JK, SSH, LBT, MS, AB, and TM: Writing – review & editing.

## CONFLICT OF INTEREST

JK is listed as inventor of a patent on AAV‐BR1, held by Boehringer Ingelheim International GmbH (patent no. US10696717B2). There are no other conflicts of interest to declare.

## Supporting information


Table S1
Figures S1‐S2

## Data Availability

The data that support the findings of this study are available from the corresponding author upon reasonable request.
