# Supplementary material for: A novel strategy for delivering Niemann‐Pick type C2 proteins across the blood–brain barrier using the brain endothelial‐specific AAV‐BR1 virus
Source: J Neurochem. 2022 May 25;164(1):6–28. doi: 10.1111/jnc.15621 (PMC10084444; doi:10.1111/jnc.15621)
Supplement: Supplementary file 1 — Table S1 Figures S1‐S2 [file JNC-164-6-s001.pdf]

A novel strategy for delivering Niemann-Pick type C2 proteins across the blood-brain barrier using the brain endothelial-specific AAV-BR1 virus

Charlotte Laurfelt Munch Rasmussen[#1], Eva Hede[#1], Lisa Juul Routhe[1], Jakob Körbelin[2], Steinunn Sara Helgudottir[1], Louiza Bohn Thomsen[1], Markus Schwaninger[3], Annette Burkhart[$1*], Torben Moos[$1*]

SUPPLEMENTAL INFORMATION

FIGURES

Figure S1

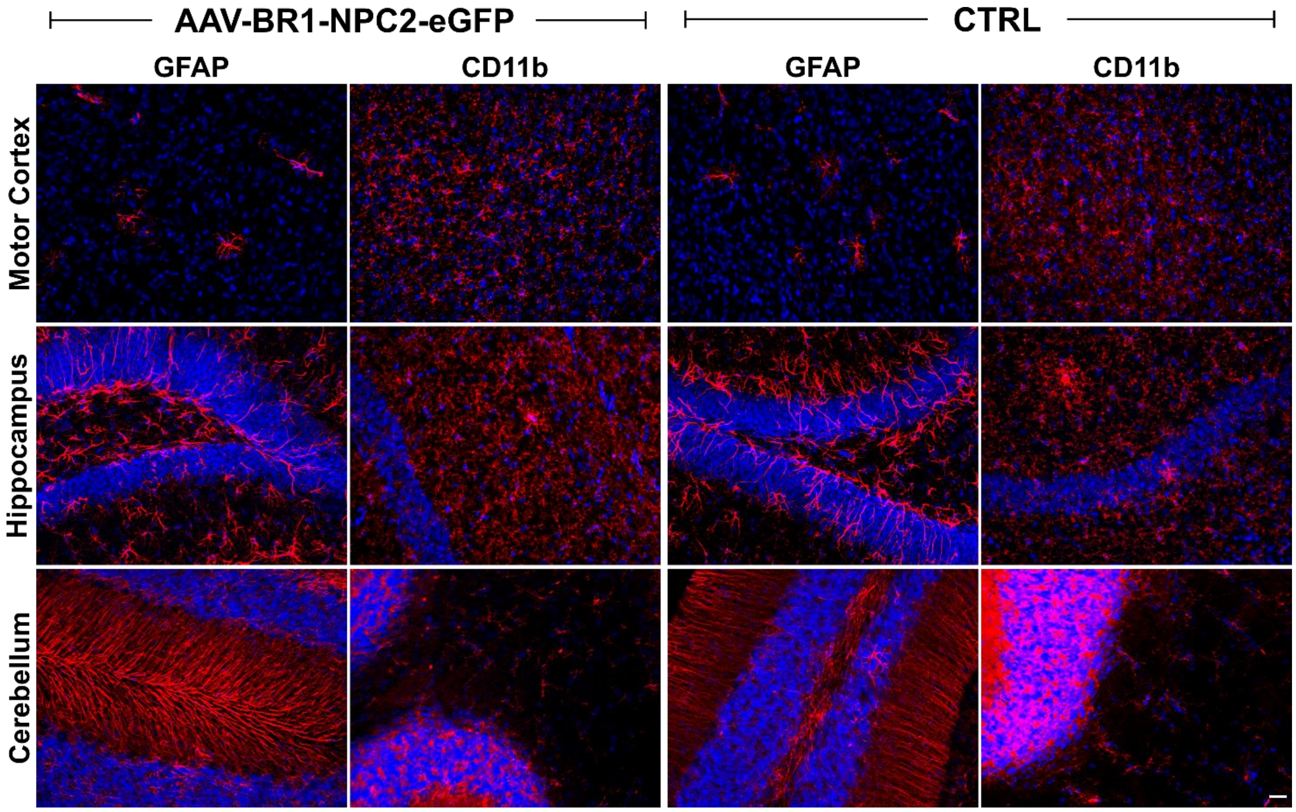

Figure S1: **Evaluation of neuroinflammation 8 weeks post-injection with AAV-BR1-NPC2-eGFP in mice compared to controls (CTRL)**. Brain sections were immunolabelled for the astrocyte marker, glial fibrillary acidic protein (GFAP), and the microglial marker, cluster of differentiation molecule 11B (CD11b) and compared to CTRL animals. GFAP positive astrocytes and CD11b positive microglial cells are seen in all brain regions. No differences in their reactivity are observed between the virus-injected mice and CTRL. The images are representative of CTRL (n=7) or AAV-BR1-NPC2-eGFP (n=6). Nuclei are counterstained with DAPI (blue). Scale bar 25µm.

Figure S2

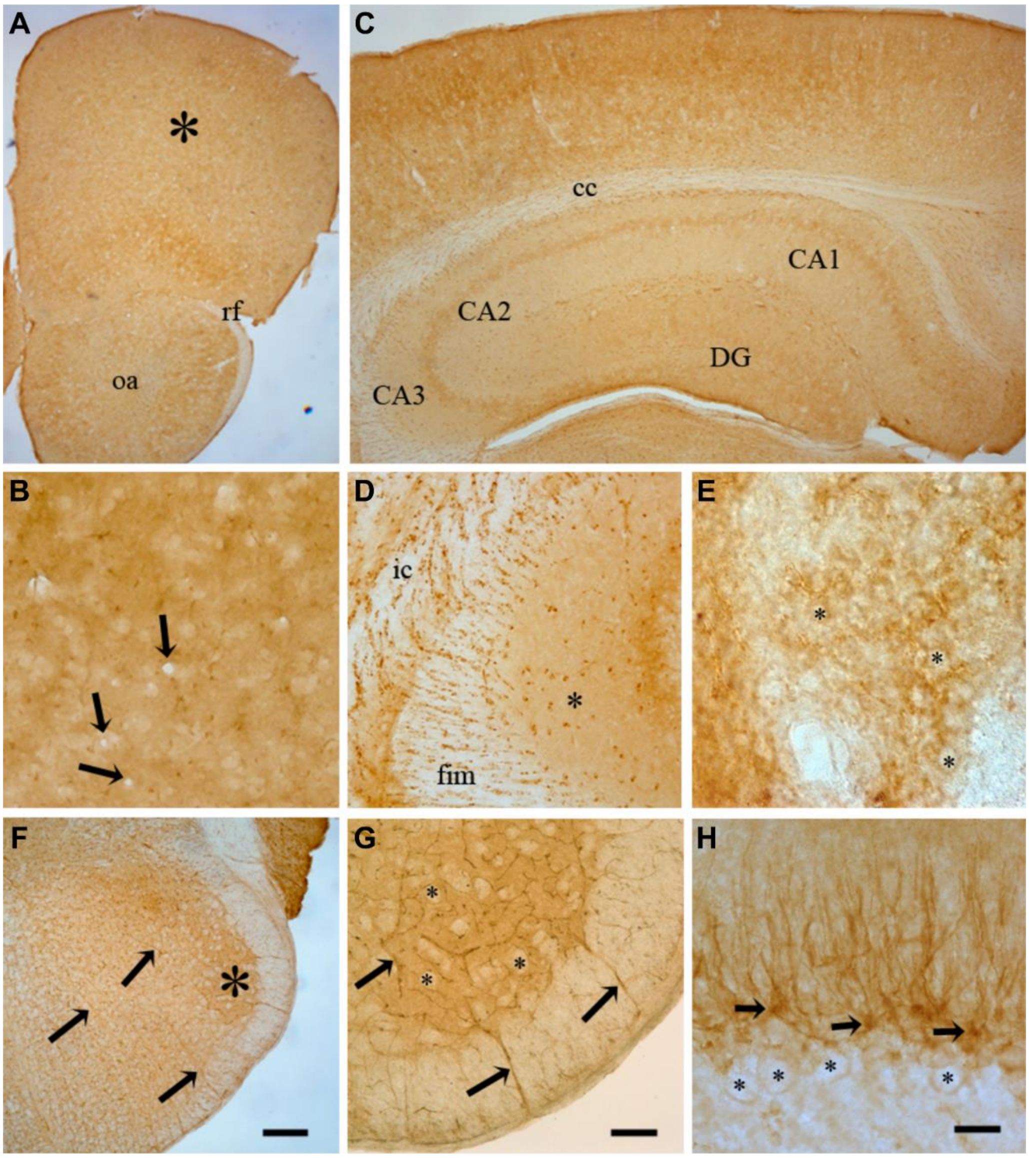

Figure S2. Distribution of endogenous NPC2 in the normal adult mouse brain. **(A)** Section from the anterior portion of the mouse brain containing the prefrontal cortex (top) and olfactory area (oa) separated from the rhinal fissure (rf). Labeling is seen in non-neuronal cells, mainly oligodendrocytes, and virtually absent from brain endothelial cells (BECs). **(B)** Part of the prefrontal cortex taken from the area marked with a large asterisk in **(A)** and shown at higher magnification showing unlabeled brain capillaries (arrows). **(C)**. Section of the forebrain containing the parietal cortex and hippocampus. CA1-CA3, fields of the hippocampus. DG, dentate gyrus. cc, corpus callosum. Labeling is mainly seen in oligodendrocytes of the white matter. **(D)** When shown in higher magnification, the labeling of the white matter is attributed to interfascicular oligodendrocytes. Labeled oligodendrocytes are also in the grey matter of the hippocampus (asterisk). fim, fimbria, ic, internal capsule. **E)** Section showing choroid plexus epithelial cells of the third ventricle. Small asterisks mark unlabeled nuclei of the choroid plexus epithelial cells. **F)** Section from the lower brainstem showing NPC2 in BECs. Arrows identify labeled capillaries, and the area indicated by the large asterisk is shown at larger magnification in **(G)** in where arrows show labeled capillaries in the region containing the facial nucleus. Small asterisks mark unlabeled nuclei of the facial neurons. **(H)** NPC2 labeling of the cortex cerebelli. Labeling is seen in Bergmann glia (arrows), whereas Purkinje cells (small asterisks) are unlabeled. Scale bars: A,C,F = 500 µm (shown in F), D,G = 50 µm (shown in G), B,E,H = 20 µm (shown in H).

TABLE

Supplemental Table 1: Calculation of effect size and sample size using the statistical power analysis G*Power (version 3.1.9.2)

**α**	**Power: 1-β**	**Mean group 1**	**SD group 1**	**Mean group 2**	**SD group 2**	**Effect size (cohens *d*)**	**Sample size**
0.05	0.8	134.3	18.33	125.1	24.83	0.42	90

Group 1: NPC2 protein concentration in cerebrum from AAV-BR1-NPC2-eGFP injected mice, group 2: NPC2 protein concentration in cerebrum from control mice (PBS-injected)

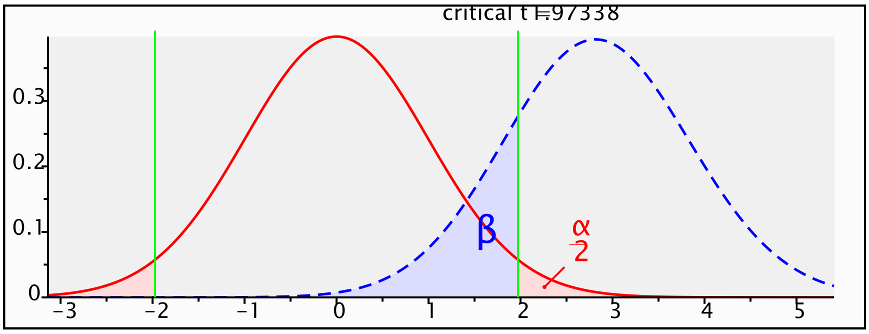

**t tests** - Means: Difference between two independent means (two groups)			
**Analysis:**	A priori: Compute required sample size		
**Input:**	Tail(s)	=	Two
	Effect size d	=	0.4215667
	α err prob	=	0.05
	Power (1-β err prob)	=	0.8
	Allocation ratio N2/N1	=	1
**Output:**	Noncentrality parameter δ	=	2.8279554
	Critical t	=	1.9733809
	Df	=	178
	Sample size group 1	=	90
	Sample size group 2	=	90
	Total sample size	=	180
	Actual power	=	0.8030887
